# C-Jun N-terminal kinase (JNK) pathway activation is essential for dental papilla cells polarization

**DOI:** 10.1371/journal.pone.0233944

**Published:** 2021-03-26

**Authors:** Jiao Luo, Xiujun Tan, Ling Ye, Chenglin Wang

**Affiliations:** 1 State Key Laboratory of Oral Diseases & National Clinical Research Center for Oral Diseases & Department of Cariology and Endodontics West China Hospital of Stomatology, Sichuan University, Chengdu, Sichuan, China; 2 Department of Endodontics, College of Stomatology, Chongqing Medical University, Chongqing, China; Università degli Studi della Campania, ITALY

## Abstract

During tooth development, dental papilla cells differentiate into odontoblasts with polarized morphology and cell function. Our previous study indicated that the C-Jun N-terminal kinase (JNK) pathway regulates human dental papilla cell adhesion, migration, and formation of focal adhesion complexes. The aim of this study was to further examine the role of the JNK pathway in dental papilla cell polarity formation. Histological staining, qPCR, and Western Blot suggested the activation of JNK signaling in polarized mouse dental papilla tissue. After performing an *in vitro* tooth germ organ culture and cell culture, we found that JNK inhibitor SP600125 postponed tooth germ development and reduced the polarization, migration and differentiation of mouse dental papilla cells (mDPCs). Next, we screened up-regulated polarity-related genes during dental papilla development and mDPCs or A11 differentiation. We found that Prickle3, Golga2, Golga5, and RhoA were all up-regulated, which is consistent with JNK signaling activation. Further, constitutively active RhoA mutant (RhoA Q63L) partly rescued the inhibition of SP600125 on cell differentiation and polarity formation of mDPCs. To sum up, this study suggests that JNK signaling has a positive role in the formation of dental papilla cell polarization.

## Introduction

At early stages, dental papilla cells appear small with a central nucleus and a few organelles. In a later phase, those cells differentiate into preodontoblasts that are located along the basement membrane (BM), and polarizing odontoblasts, which are tall columnar with a polarized distribution of nuclear and cytoplasmic organelles [1, 2]. It is generally believed that the dental papilla cells and their polarization contributes to tooth formation, secretion of dentin, and pulp repair; yet, the molecular mechanism remains poorly understood.

So far, only a few studies explored the molecular mechanism during the process of dental papilla cell polarization. For example, Jiménez *et al* found that defective odontoblast polarization, such as apical nuclei distribution, was found in epiprofin (-/-) mice [3]; epiprofin is a transcription factor that regulates dental epithelial cell proliferation and is essential for ameloblast and odontoblast. Moreover, Choi *et al* discovered that dentin formation was disrupted in Distal-Less 3 (DLX3)-mutant mice [4]; DLX3 is an autosomal dominant condition characterized by anomalies in bone, hair, and teeth.

MAP1B [5], PRICKLEs [6], and Celsr1 [7] have been considered as essential polarity proteins localized in the odontoblastic layer C-Jun N-terminal kinase (JNK) kinase, which affects the spine morphology [8], regulates the expression of polarity complex genes Celsr2, Numb, Prkcz, Llgl2 in bone marrow hematopoietic progenitor cells [9]. Furthermore, previous studies have shown that JNK signaling participates in cell proliferation, differentiation, apoptosis, and cytoskeleton reorganization [10–12], and regulates plane cell polarity (PCP) [13]. Our previous study [14] found that the JNK pathway regulates human dental papilla cell adhesion, migration, and formation of focal adhesion complexes. In this study, we further examined the role of the JNK pathway in dental papilla cell polarity formation.

## Materials and methods

### Tissue samples collection

All procedures performed in this study were in accordance with the recommendations included in the Guide for the Care and Use of Laboratory Animals of the National Institutes of Health. The Animal Care and Ethics Committee of West China School of Stomatology, Sichuan University approved the experiment protocol.

ICR/CD1 mice were housed in small groups in specific pathogen-free conditions at 22°C with a 12h light/ dark cycle and ad libitum access to food and water. The pregnant mice were culled using CO2 inhalation after successful mating for some days. Cardiac perfusion with saline was performed before cervical dislocation and collection of tooth germ tissue. The heads or mandibles with tooth germs were isolated, at embryonic day 17.5 (E17.5), E18.5 (vaginal plug = E0.5), and postnatal day 1 (P1). All samples were fixed in 4% paraformaldehyde (PFA), embedded in paraffin, and cut into 5μm sections. Hematoxylin and eosin(H&E) staining and immunofluorescence (IF) or immunohistochemistry (IHC) staining were then performed.

### Immunofluorescence and immunohistochemistry

The slides were dewaxed in xylene, rehydrated, and then subjected to antigen retrieval in citrate buffer at 99°C for 5 min×3. For IF, the primary antibody anti-GM130 (Golgi matrix protein of 130kDa) (1:10, BD Pharmingen, USA) was incubated overnight at 4°C. Nuclear was stained using DAPI. For IHC, the primary antibodies, anti-phospho-JNK (Tyr183/Tyr185) or anti- JNK (1:50, Cell Signaling Technology Inc., MA, USA) were incubated overnight at 4°C; next procedures were performed with SP9001 kit and DAB Staining Kit (ZSGB-Bio, Beijing, China).

### Quantitative real-time PCR and Western bolt

Before RNA extraction and protein extraction, E17.5 and P1 tooth germ were separated into mesenchymal and epithelial tissue; the separation process was performed as previously described [15].

Total RNA was isolated from E17.5, P1 separated tooth epithelial, and mesenchymal tissues using TRIzol Reagent (Life Technologies, Carlsbad, CA, USA) according to manufacturer’s protocol. Next, cDNA was synthesized using the PrimeScript RT Reagent Kit (Takara, Dalian, China) and measured using a CFX96^™^ real-time PCR detection system (Bio-Rad, USA) as previously described [16]. Primer sequences were searched from PrimerBank (S1 Table). Relative gene expression levels are presented as the mean and standard deviation from three independent experiments.

Total proteins of E17.5 and P1 tooth germ mesenchymal tissues were isolated, measured, transferred to a membrane, and incubated with anti-GAPDH, anti-phospho-JNK (Tyr183/Tyr185) (1:1000), anti-GM130 (1:500), and HRP-conjugated anti-rabbit secondary antibodies (1:1000, Cell Signaling Technology Inc., MA, USA). Finally, we determined the ratio of the target protein to the reference protein. The obtained ratios are presented as the mean and standard deviation from three independent experiments.

### Tooth germ organ culture *in vitro*

Mandibular first molar tooth germs from E16.5 CD1 mouse embryos were harvested under a dissection microscope, following conventional procedures [15, 17]. Separated tooth germs were then cultured in modified Trowell-type organ culture with a 12- well 8.0 μm Transwell chamber (BD Biosciences, USA) and grown at the air-liquid interface [18–20], with or without 15μM SP600125 (Sigma, St. Louis, MO, USA) in a cultured medium. For concentration screening, E16.5 tooth germs were cultured with 10μM or 15μM SP600125 for 48hr before protein collection and western blot analysis, and results shown that 15μM could significantly inhibit the expression of P-JNK in tooth germs. The culture plates were kept in a humidified incubator at 37°C in 5% CO2 for 5 or 7 days; the medium was changed every two days. Consequently, tooth germs were rinsed and fixed in 4% ice cold-PFA, embedded in paraffin, and cut into 5μm sections. H&E staining was performed to examine tissue and cell morphology.

### Cell culture and JNK signaling inhibition

The mouse dental papilla cells (mDPCs) were cultured from separated dental papilla tissues of P1 tooth germs following digestion with 3 mg/ml collagenase I (Sigma, St. Louis, MO, USA) for approximately 45 min at 37°C. The tissue piece and cell suspension were seeded into 60-mm cell culture dishes and cultured in Dulbecco’s modified Eagle’s medium (DMEM, Corning, USA) supplemented with 15% fetal bovine serum (FBS, Gibco, USA) and 1% penicillin/streptomycin (P/S, Hyclone, USA). Upon 90% confluence, the mDPCs were collected and sub-cultured for use. Mouse odontoblast-like A11 cells line was maintained in DMEM supplemented with 10% FBS and 1% P/S at 37°C and 5% CO2.

For concentration screening, cells were cultured with 5μM, 10μM or 15μM SP600125 for 48 hr before protein collection and western blot analysis, and results shown that 10μM could significantly inhibit the expression of P-JNK in cells. MDPCs and A11 cells were seeded at the same density and pre-incubated with 10μM SP600125 for 2h at 37°C before scratch assay or 24h before osteogenic medium (OM) treatment; OM contained high-glucose DMEM supplemented with 10% FBS, 50μg/mL ascorbic acid, 10 mmol/L b-glycerol phosphate, 10 nmol/L dexamethasone, 2 mmol/L L-glutamine (Sigma-Aldrich, St. Louis, MO, USA), 100 U/mL penicillin and 100 mg/mL streptomycin (GIBCO-BRL, Life Technologies, Grand Island, NY, USA). Medium, inhibitor, and osteogenic medium were freshly added every second day.

### Scratch assay and cell immunofluorescence

The scratch assay and cell IF staining were performed as previously described [14, 21, 22]. Briefly, cells were treated with 10μM SP600125. After 2h treatment, a line was then drowned using a marker on the bottom of the dish, and then a sterile 200-μl pipet tip was used to scratch separate wounds through the cells, moving perpendicular to the line. After 24h, the cells were gently rinsed twice with PBS to remove floating cells and analyzed using an inverted microscope (Diaphot, Nikon Corporation, Tokyo, Japan). Relative rates of cell migration were measured and expressed as a percentage of the initial length at zero time.

Next, fixed cells were incubated with the primary antibodies, anti-GM130(1:10), and the secondary antibody, anti-rabbit IgG FITC conjugated (1:200, Santa Cruz, CA, USA). Cells at the leading edge of the scratch line were observed, with anti-GM130 labeled Golgi apparatus distribution. Quantitation of cells at the leading edge with Golgi polarized within a 120°arc in front of the nucleus was analyzed. Each experiment was repeated three times.

### Alkaline phosphatase (ALPase) and alizarin red S (ARS) staining

In this study, we used ALPase staining and ARS staining to check the change of cell differentiation of mDPCs and A11 cells. For ALPase staining, cells were cultured in OM for 3 (A11 cell) or 5 (mDPCs) days with or without 10μM SP600125 and tested by ALPase staining kit (Beyotime Institute of Biotechnology, Hai-men, China) according to manufacturer’s instructions. For ARS staining, cells were cultured in OM for 7(A11 cell) or 14(mDPCs) days with or without 10μM SP600125 and tested by ARS staining (Sigma-Aldrich) according to manufacturer’s instructions. Each experiment was repeated three times.

### Polarity-related gene screening

Total RNAs were isolated from E17.5 and P1 dental papilla tissue, from mDPCs before and 6h after OM induction, from A11 cells before and 6h after OM induction. Then cDNA was synthesized and real-time PCR was performed as described above. Primers of some polarity-related genes are shown in S1 Table.

After screening out some up-regulated polarity-related genes during the process of dental papilla development and mDPCs or A11 differentiation, the expression of these screened genes was confirmed in SAPK/JNK siRNA transfected or SP600125 treated mDPCs. For siRNA transfection, the cells were transfected with 100nM SAPK/JNK siRNA I or control siRNA (Cell Signaling Technology Inc., MA, USA) by using Lipofectamine 3000 (Invitrogen, Life Technologies, USA) according to the manufacturer’s instructions. For SP600125 treatment, the cells were incubated with 10μM SP600125 for 24h at 37°C. Then RNAs were isolated and qPCR was performed as above.

### RhoA mutant adenovirus construction and transfection

In our previous study [14], we constructed RhoA mutant recombinant adenovirus including Ad-RhoA WT (wild type RhoA) and Ad-RhoA Q63L (constitutively active RhoA mutant), and found that both RhoA and JNK signaling participate in regulating cell adhesion and migration. In this study, RhoA was further chosen to examine its role in JNK signaling-regulated cell polarity. Briefly, mDPCs were transfected by Ad-RhoA WT or Ad-RhoA Q63L for 48h. Before ALPase staining as above, mDPCs were cultured in OM for 5 days with or without 10μM SP600125. Before scratch assay and IF staining, mDPCs were cultured in a regular medium for 24h with or without 10μM SP600125. Each experiment was repeated three times.

### RhoA pull-down assay

Ad-RhoA WT or Ad-RhoA Q63L was transfected into mDPCs for 48 h, following SP600125 treatment for 24 h. GST pull-down assay with a glutathione transferase (GST) fusion protein containing the RhoA binding domain of rhotekin (rhotekin-GST) was performed by the manufacturer’s protocol using GTPase Pull-Down kit (Thermo Scientific, Waltham, MA, USA). Activated and total RhoA were detected by Western blot analysis using the anti-RhoA antibody [14].

### Statistical analysis

All values were calculated as the mean ±standard deviation (SD). The statistical significance was determined by unpaired Student’s t-test of variance at *P*<0.05.

## Results

### Dental papilla cells polarization during tooth germ development

**Fig 1:**
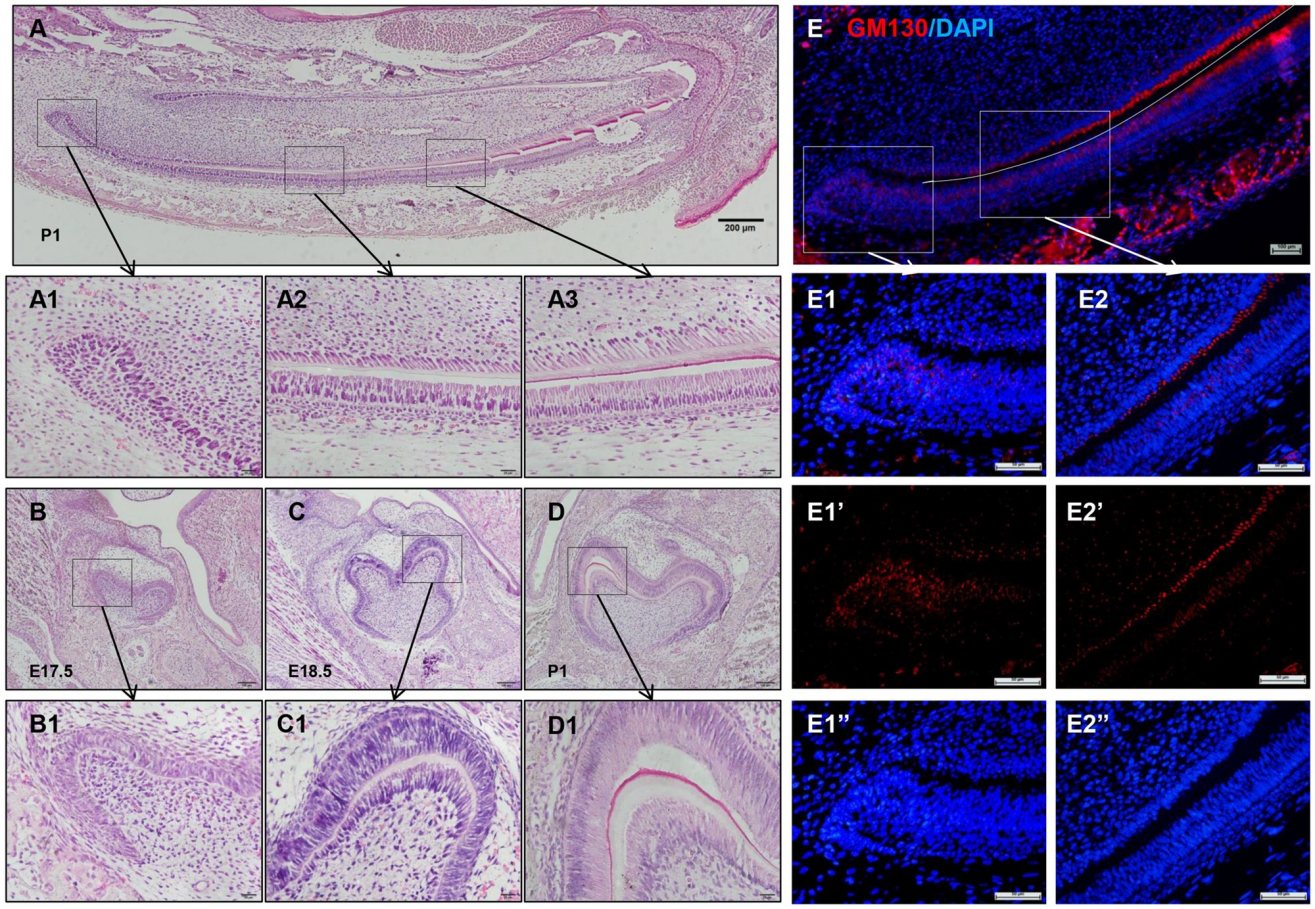
Morphogenesis and function of dental papilla cell polarization. (A) H&E staining of P1 incisor. From apical to the coronal region: A1, proliferation zone. A2, differentiation zone. A3, secretory zone. (B-D) H&E staining of the mandibular first molars from E17.5, E18.5 and P1. B1, C1 and D1, at the cusps region, dental papilla cells changed from a flat shape to a columnar with polarity formation. (E) IF of GM130 at P1 incisor. GM130 was laterally distributed in the functional side of odontoblasts (E2, E2’, E2”), but not in the cervical loop region (E1, E1’, E1”). E: embryonic, P: postnatal, Bars: 200μm (A), 20μm (A1-A3, B1-D1), 100μm (E), 50μm(E1-E2”).

At incisor, dental papilla cells were small and undifferentiated in the proliferation zone (Fig 1A1), and columnar with a polarized distribution of the nucleus in the differentiation zone (Fig 1A2). In addition, functional odontoblasts with matrix synthesis and secretion functions were observed in the secretory zone (Fig 1A3). The same phenotype was observed at the cusp region of the first molar (Fig 1B–1D).

Dental papilla cells showed different morphology at different stages. In addition, IF staining showed that Golgi marker GM130 was also gradually and orderly distributed at the functional side close to BM in differentiation and secretory zone, thus suggesting cell polarity formation in these zones (Fig 1E). GM130 was laterally distributed in the functional side of odontoblasts (Fig 1E2, 1E2’ and 1E2”), but not in the cervical loop region (Fig 1E1, 1E1’ and 1E1”).

### JNK signaling was activated in polarized dental papilla cells

**Fig 2:**
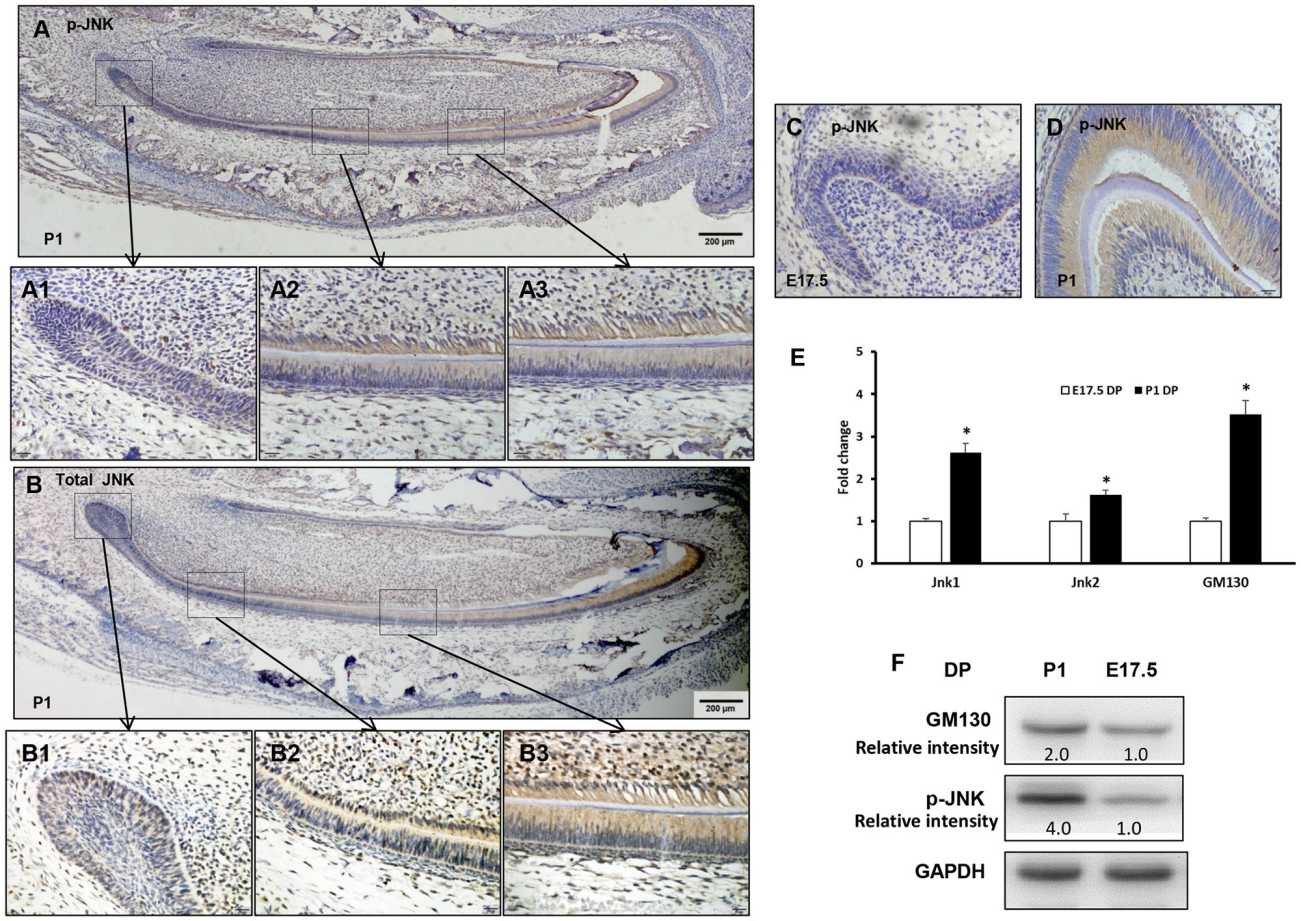
Activation of JNK signaling in polarized dental papilla cells. (A) At P1 incisor, immunochemistry revealed that p-JNK was mainly expressed in the differentiation zone (A2) and secretory zone (A3), but not in the proliferation zone (A1). (B) Total JNK was widely expressed in odontogenic cells (B1, B2, B3). (C) At E17.5 first molar, p-JNK was barely visible in the dental papilla. (D) At P1 first molar, p-JNK was strongly expressed in odontoblasts. (E) RT-PCR showed increased expression of Jnk1, Jnk2 and GM130 in P1 dental papilla tissue, compared with E17.5. (F) Western blot showed increased p-JNK and GM130 in P1 dental papilla tissue, compared with E17.5. DP: dental papilla, Bars: 200 μm (A,B), 20 μm (A1-A3,B1- B3,C,D).

In P1 incisor, p-JNK was mainly expressed in differentiated and secretory odontoblasts (Fig 2A1–2A3), while total JNK was widely expressed in odontogenic cells (Fig 2B1–2B3). In molar, p-JNK was barely visible in dental papilla at E17.5 (Fig 2C), but it was strongly expressed in odontoblasts at P1 (Fig 2D). The results showed that the spatial and temporal expressions of the JNK signal were positively correlated with dental papilla cell polarization.

Dental epithelium and dental papilla were separated from the mandibular first molars of E17.5 and P1 (S1A and S1B Fig), and the expression of epithelium marker Cdh1 and mesenchymal marker Lhx8 verified the source properties and availability of separated tissue (S1C Fig). Compared with E17.5, increased expression of Jnk1, Jnk2, and GM130/Golga2 were found in the P1 dental papilla (Fig 2E and 2F). These results suggested that JNK signaling is activated with dental papilla cell polarity.

### JNK inhibitor retarded tooth germ development *in vitro*

**Fig 3:**
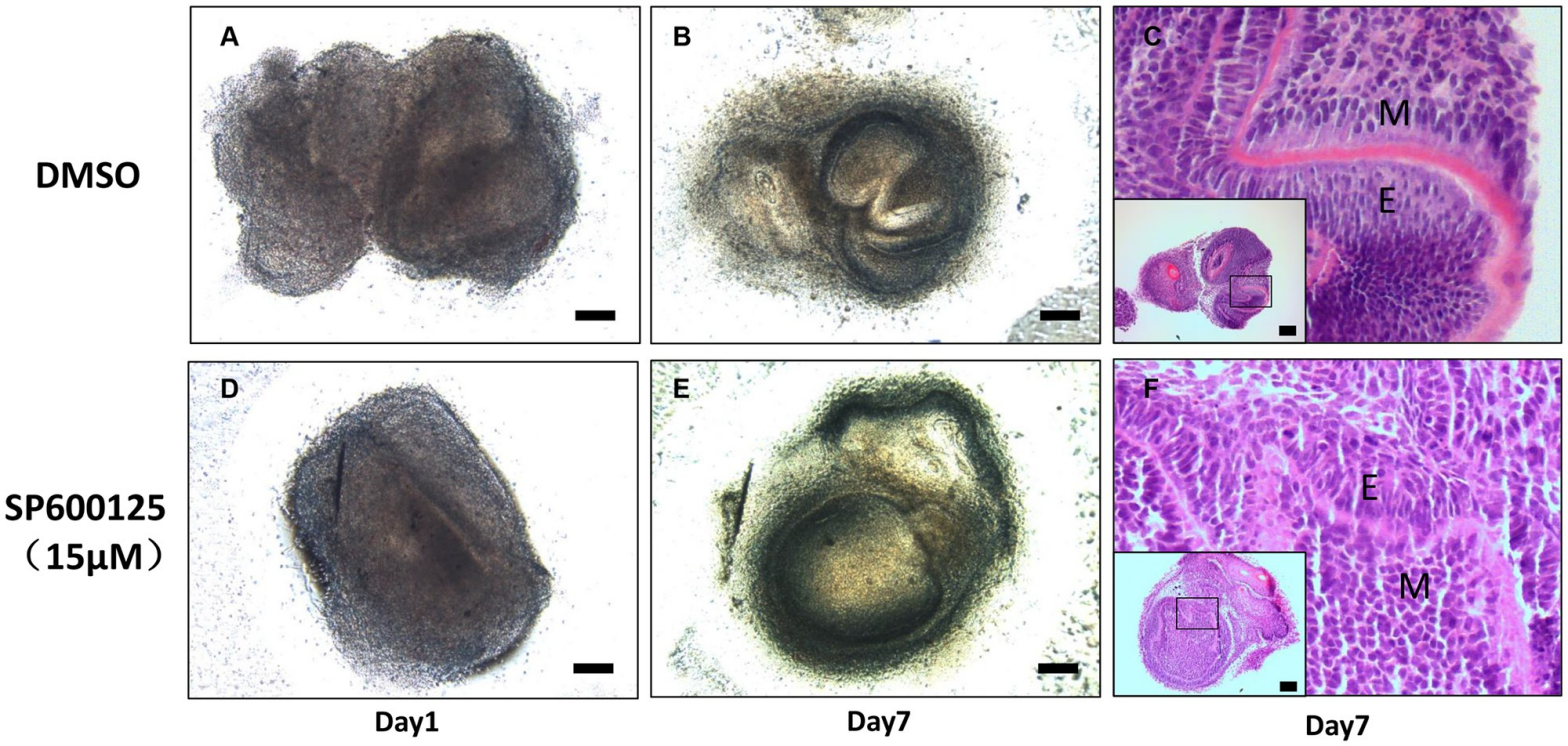
SP600125 postponed tooth germ development *in vitro*. (A, D) Organ culture with DMSO or SP600125(15μM) for 1 day, a whole view of E16.5 tooth germs. (B, E) Organ culture with DMSO or SP600125(15μM) for 7 days; the whole view of E16.5 tooth germs. (C) H&E staining of the sample with DMSO showed the development stage of tooth germ as late bell stage, with plenty of polarized odontoblasts along with BM. (F) H&E staining of the sample with SP600125 (15μM) showed the development stage of tooth germ as early bell stage, with undifferentiated mesenchymal cells along with BM. Bars = 200μm. M: mesenchyme, E: epithelium.

Given JNK activation has a role in tooth germ development, we wondered whether JNK inhibitor SP600125 could induce the retardation of tooth development. Concentration screening showed that 10μM SP600125 was a proper concentration for cell culture (S2A Fig), and 15μM SP600125 was suitable for tooth germ culture (S2B and S2C Fig). Briefly, isolated E16.5 tooth germs were cultured in the air-liquid interface culture system for 7 days. Tooth germ development was delayed with SP600125 treatment, especially the formation of the enamel organ and dental papilla. For example, the shape of the enamel organ treated with SP600125 was more obscure than one with DMSO (Fig 3A, 3B, 3D and 3E). Moreover, H&E staining of the sample with DMSO showed the development stage of tooth germ as late bell stage, with plenty of polarized odontoblasts along with BM. Nevertheless, a sample treated with 15μM SP600125 showed the development stage of tooth germ as early bell stage, with undifferentiated mesenchymal cells along with BM (Fig 3C and 3F).

### SP600125 inhibits migratory and functional cell polarity

**Fig 4:**
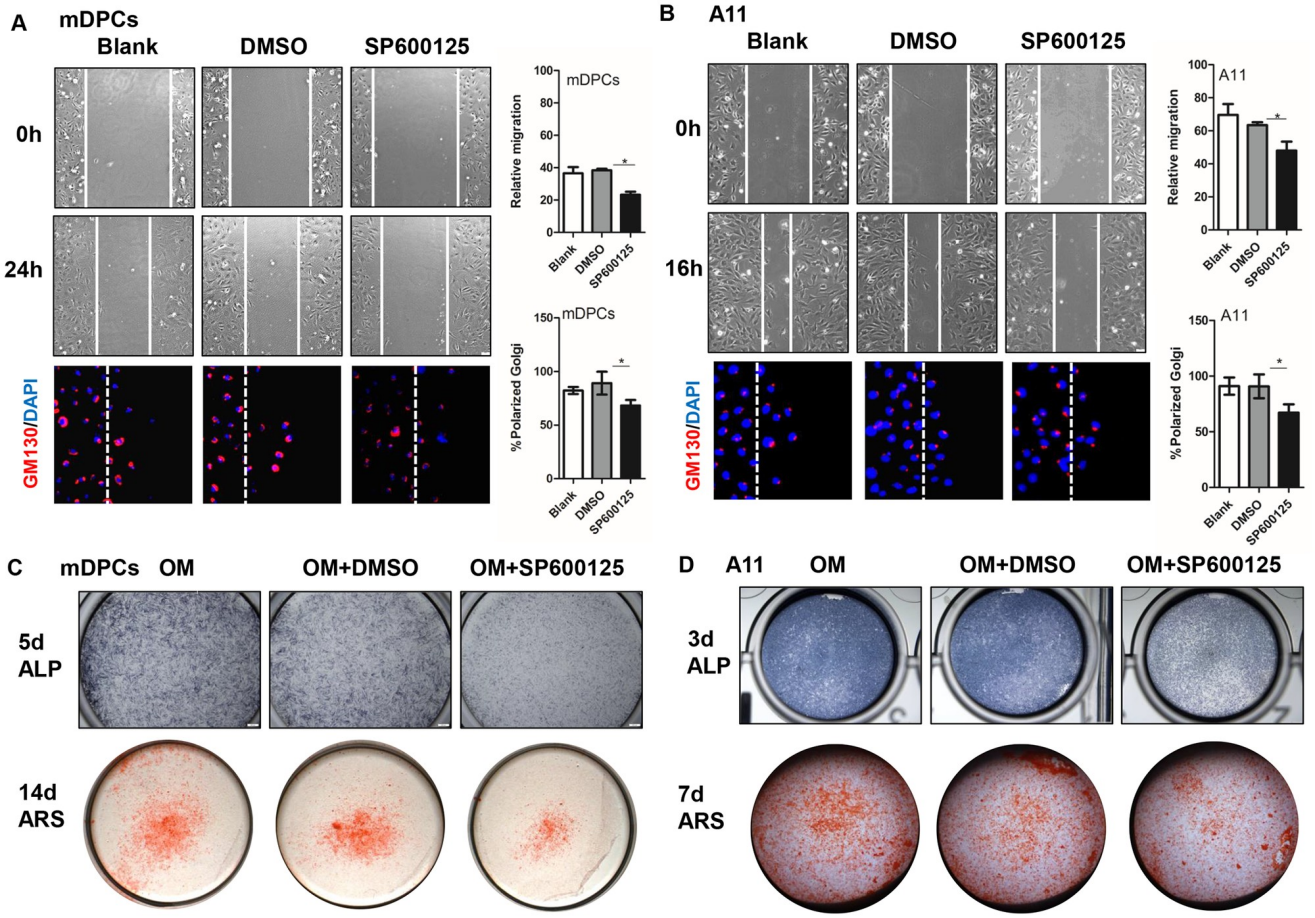
SP600125 reduced cell migration and differentiation. Scratch assay and cell immunofluorescence of GM130 in mDPCs (A) or A11 cells (B) showed that 10μM SP600125 could inhibit cell migration and polarity formation along the scratch line. ALP staining and alizarin red staining in mDPCs (C) or A11 cells (D) in OM with 10μM SP600125 treatment showed that SP600125 treatment could inhibit cell differentiation. Bars: 50μm (A,B), 200μm (C,D) *P<0.05 with significance.

As tooth development and cell polarity formation were retarded with SP600125 treatment, we wondered if the migration and differentiation ability of dental papilla cells with SP600125 treatment was changed. With SP600125 treatment, the migration of mDPCs and A11 cells was reduced, and the number of cells at the leading edge with the Golgi polarized within a 120°arc in front of the nucleus were decreased (Fig 4A and 4B). Moreover, ALPase activity was significantly reduced in OM-induced mDPCs on day 5 and A11 cells on day 3. Mineralized nodules were decreased at day 14 and 7, respectively (Fig 4C and 4D). These results suggested that JNK signaling participates in cell migration and differentiation, and SP600125 treatment inhibits the migratory polarity and functional polarity of mDPCs or A11 cells.

### Some polarity-related genes participate in JNK signaling regulated development of dental papilla tissues and cell differentiation of dental papilla cells

**Fig 5:**
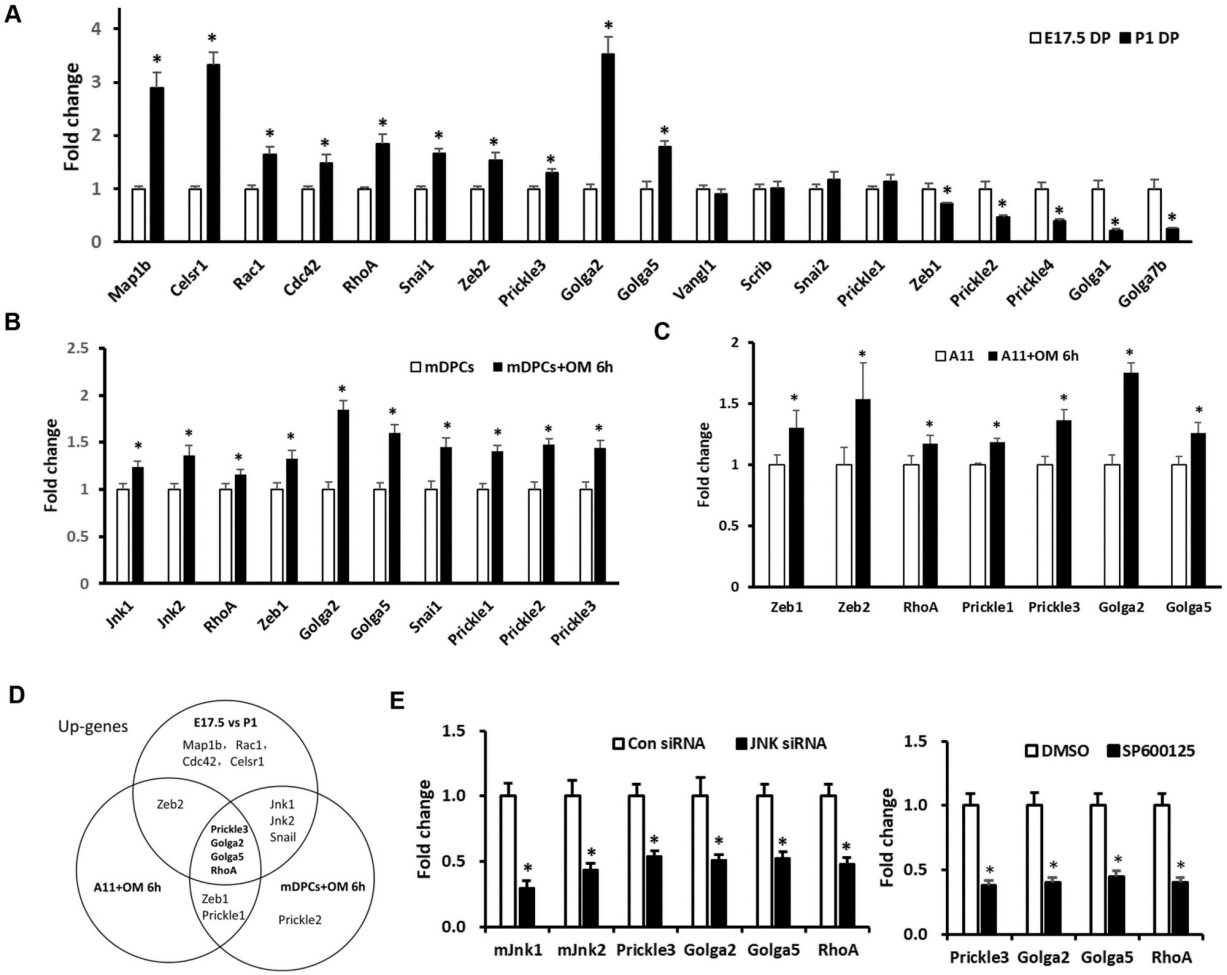
Polarity-related gene expression in dental papilla tissues and cells. (A) Expression of polarity-related genes in P1 dental papilla tissues compared with E17.5. (B) Up-regulated screened genes in mDPCs after osteogenic induction for 6h. (C) Up-regulated screened genes in A11 cells line after osteogenic induction for 6h. (D) Up-regulated genes both in A, B, and C. (E) Expression of Prickle3, Golga2, Golga5, and RhoA in mDPCs with SAPK/JNK siRNA transfection or SP600125(10μM) treatment, results showed that similar changes between JNK siRNA and SP600125 treatment. DP: dental papilla, OM: Osteogenic medium, mDPCs: mouse dental papilla cells. *P<0.05 with significance.

By gene screening, we found that some polarity-related genes as Map1b, Rac1, Cdc42, RhoA, Celsr1, Snai1, Zeb2, Prickle3, Golga2, and Golga5 were up-regulated in dental papilla tissues of P1, compared with E17.5 (Fig 5A). Meanwhile, the expression of these genes was detected in OM-induced mDPCs and A11 cells (Fig 5B and 5C). Finally, we found that Prickle3, Golga2, Golga5 and RhoA were up-regulated during the process of dental papilla development, as well as mDPCs and A11 differentiation (Fig 5D). Interestingly, their expression was accordingly reduced in mDPCs transfected with SAPK/JNK siRNA or treated with SP600125(Fig 5E). These results might suggest the possible role of Prickle3, Golga2, Golga5, and RhoA participating in the JNK signal-regulating pathway.

### RhoA signaling contributed to the JNK-regulated cell differentiation and migratory polarity formation

**Fig 6:**
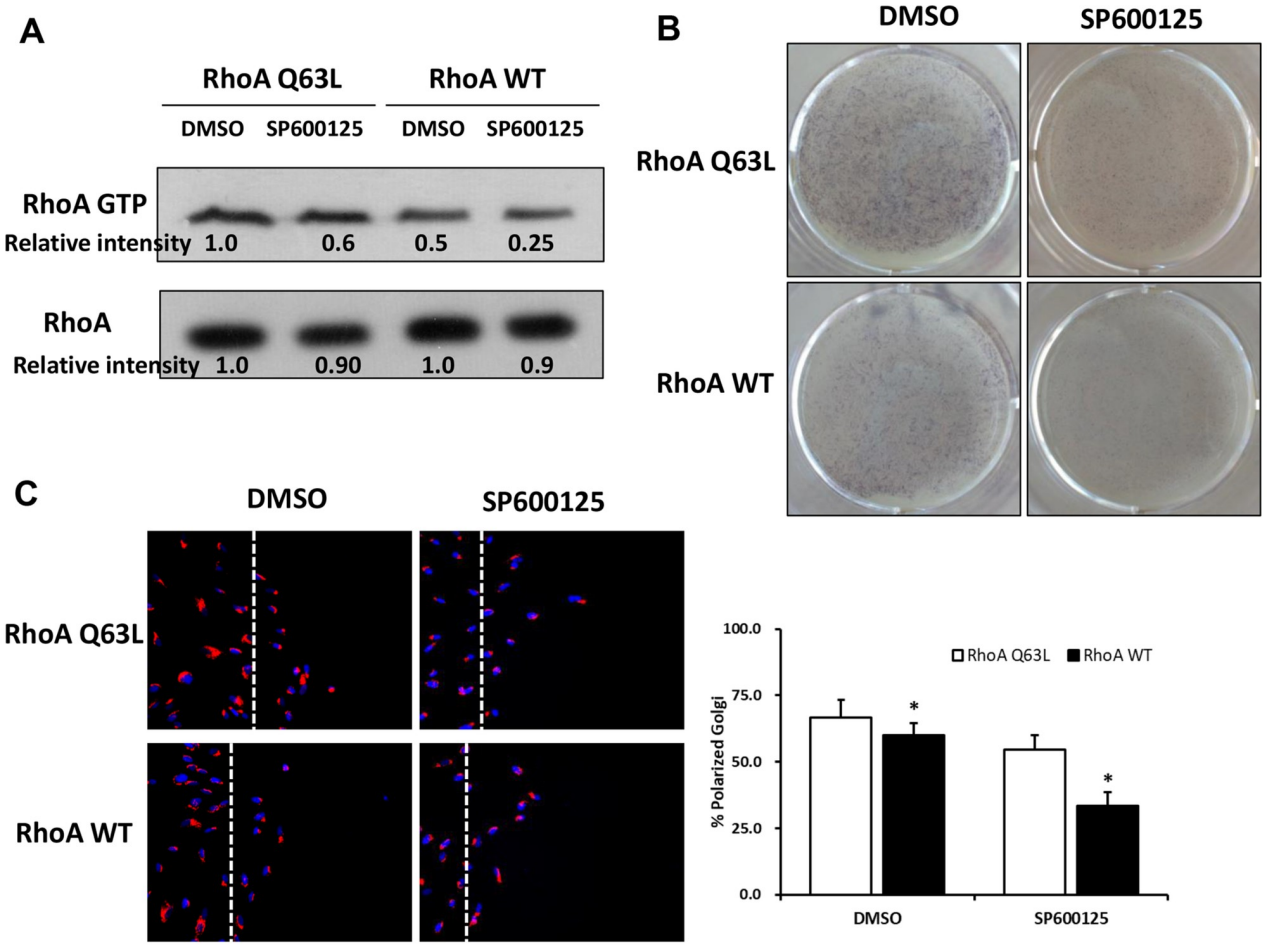
RhoA signaling contributed to the JNK-regulated cell differentiation and migratory polarity formation. First, mDPCs were infected with RhoA mutant adenoviruses (RhoA WT or RhoA Q63L) for 48h, then were cultured with DMSO or SP600125(10μM). (A) After 24 h with SP600125 treatment, GST-Pull down assays were performed, RhoA Q63L could partly up-regulate the RhoA activity with SP600125 treatment. (B) After 5 days with SP600125 treatment, ALPase staining showed that RhoA Q63L could partly rescue SP600125-regulated inhibition of mDPCs differentiation. (C) After 10 h with SP600125 treatment, cell immunofluorescence of GM130 for the cells along the scratch line were analyzed, and RhoA Q63L could partly rescue SP600125-regulated decreased of mDPCs polarity formation. *P<0.05 with significance.

In our previous study, we found that RhoA-JNK signaling pathway affects human dental papilla cell adhesion, migration, and differentiation [14]. In this study, we hypothesized that RhoA would have a role on JNK-regulated cell polarization. Thus, we chose the RhoA gene and used Ad-RhoA WT and Ad-RhoA Q63L to transfect mDPCs so as to study the role of RhoA activity on these processes in mDPCs. SP600125significantly down-regulated RhoA activity (Fig 6A, lane 1 vs. lane 2, lane 3 vs. lane 4), while RhoA Q63L up-regulated RhoA activity (Fig 6A, lane 1 vs. lane 3, lane 2 vs. lane 4). Compared to RhoA WT, RhoA Q63L significantly up-regulated SP600125-inhibited RhoA activity (Fig 6A), and partly rescued SP600125-inhibited mDPCs differentiation (Fig 6B) and SP600125-inhibited mDPCs polarity formation (Fig 6C). These data suggest that RhoA participates in JNK signaling related cell polarization, which confirmed the results of polarity-related gene screening.

## Discussion

In this study, several time points were selected during the morpho-differentiation stage; E17.5 represents the beginning of cell differentiation, and P1 at the end of the differentiation phase. Similar to tissue structure [23], no significant cell morphology changes and polarity formation are observed in E17.5 tooth germ. Contrary, cell polarity is very obvious in P1 tooth germ. In this study, we chose E16.5 tooth germ to given extraneous treatment for tooth germ organ culture, because the treatment should be given before and close to morpho-differentiation stage, in order to avoid organ culture *in vitro* for too long time. We found that E16.5 tooth germ was developed to P0 [24] or E19.5 stage tooth germ after 7 days of organ culture *in vitro*. Above all, E17.5 and P1 time points could be used to represent before and after dental papilla cells differentiated, and E16.5 tooth germ was suitable to study the change of dental papilla tissue changes by organ culture. With reference to cell study, considering the accessibility and representation, we cultured poorly differentiated mDPCs, and highly differentiated odontoblast-like cell line A11 *in vitro*; these cells, represent different differentiation status of dental mesenchymal cells.

Previous studies have demonstrated the role of JNK signaling on cell polarity and mesenchymal fate [13, 25]. Moreover, our previous study suggested that, JNK signaling had a role in odontoblasts maturation [14]. In addition, some authors [26] examined the role of JNK signaling in tissue morphology and found activated JNK along microtubules in cultured neurons from the intermediate zone. The application of SP600125 caused irregular morphology and increased stable microtubules in neurons processes, which suggested a possibility of the involvement of JNK in controlling tubulin dynamics in migrating neurons [26]. In the present study, we performed an organ culture experiment and found that JNK signaling was positively correlated with the polarity formation of dental papilla cells; while SP600125 inhibited this effect. In cell culture, SP600125 inhibited mDPCs migration and reduced cell number at the leading edge with polarized Golgi and inhibited ALPase activity and mineralized nodules formation in mDPCs and A11. The above results inspired us to further explore the polarity-related mechanism.

Usually, cell polarity is defined as asymmetry in cell shape, protein distribution, and cell function [27]. One characteristic of cell polarity is a polarized distribution of cytoplasmic organelles. As a marker of cell polarity, Golgi complex functions in a variety of membrane-membrane and membrane-cytoskeleton tethering events. GM130, a static structural matrix associated with the Golgi apparatus, is vital for protein transport, cell migration, and polarization [28, 29]. In this study, we chose GM130 to observe the polarity change. In tooth germ, IF data showed that GM130 were laterally distributed in the functional side of odontoblasts, suggesting functional cell polarity. A previous study examined cell polarity change by quantitation of cells at the leading edge with Golgi polarized within a 120° arc in front of the nucleus as previously described [14, 21, 22], suggesting migratory cell polarity. Additionally, we examined the gene expression of Golga1, Golga2, Golga5, and Golga7b, which showed that the expression of Golga2 and Golga5 were increased in P1 dental papilla compared to E17.5; yet, this was not observed for Golga1 and Golga7b. Golga5, also known as Golgin 84, is a transmembrane golgin combined with Rab1, which participates in the formation and maintenance of the Golgi apparatus [30]. In our study (Figs 4 and 5), JNK inhibitor could disturb GM130 polar distribution, and SAPK/JNK siRNA infection could also decrease the expression of Golga2 and Golga5 mRNA. Accordingly, we speculate that JNK signaling has a role in golgin expression and distribution, which further affects cell polarity formation.

Another characteristic of cell polarity is changed cell shape. In this study, Prickle3, a planar cell polarity protein, RhoA, prototypical Rho GTPases have shown to have an integral role in cytoskeletal arrangement, membrane-trafficking pathways, and ECM interactions, which are all crucial steps for cell polarization [31]. Interestingly, the expression of RhoA in our study showed the same trend as Prickle3, which has shown to be positively expressed in papillary layer cells and some singular cells adjacent to the dentin [6] In our previous study, we examined the role of RhoA on the migration of human dental papilla cells using RhoA mutant adenoviruses and found that RhoA activity could active JNK signaling [14]. In the present study, JNK inhibitor treatment down-regulated the expression of RhoA mRNA and inhibited RhoA activity. After SP600125 treatment, constitutively active RhoA (RhoA Q63L) could partly rescue SP600125-regulated inhibition of mDPCs polarity formation and differentiation. This result suggested that RhoA signaling contributes to the JNK-regulated cell differentiation and polarity formation of mDPCs. Yet, the role of Prickle3, Golga2, Golga5 in JNK-regulated cell polarity formation, and differentiation needs to be further investigated.

## Conclusion

JNK signaling has a positive role in dental papilla cell polarization. JNK can also regulate dental papilla cell polarity distribution and cell migration, which in turn affect cell differentiation, protein synthesis, and secretion. Moreover, our data suggested that Prickle3, Golga2, Golga5, and RhoA are closely associated with the mechanism of polarization. Besides, RhoA signaling contributes to JNK-regulated cell differentiation and polarity formation.

## Supporting information

S1 Raw images (PDF)

S1 Fig Separated dental epithelium and papilla tissue. (A, B) Separated dental epithelium and dental papilla under the stereomicroscope. (C) Expression of epithelium marker Cdh1 and mesenchymal marker Lhx8 in the separated dental papilla. DP: dental papilla, DE: dental epithelium.
(TIF)

S2 Fig Dose-response experiment of SP600125 on cell culture and tooth germ organ culture *in vitro*. (A) For mDPCs culture, 10μM SP600125 could inhibit JNK signaling activation after 48 hr. (B) For E16.5 tooth germ culture, 15μM SP600125 could inhibit JNK signaling activation after 48 hr. (C) For E16.5 tooth germ culture, 25μM SP600125, but not 15μM SP600125, has some toxic effect on the tissues after 5 days. Bars = 200μm.
(TIF)

S1 Table Primer sequences from Primerbank. (DOCX)
