# Supplementary material for: C-Jun N-terminal kinase (JNK) pathway activation is essential for dental papilla cells polarization
Source: PLoS One. 2021 Mar 26;16(3):e0233944. doi: 10.1371/journal.pone.0233944 (PMC7996994; doi:10.1371/journal.pone.0233944)

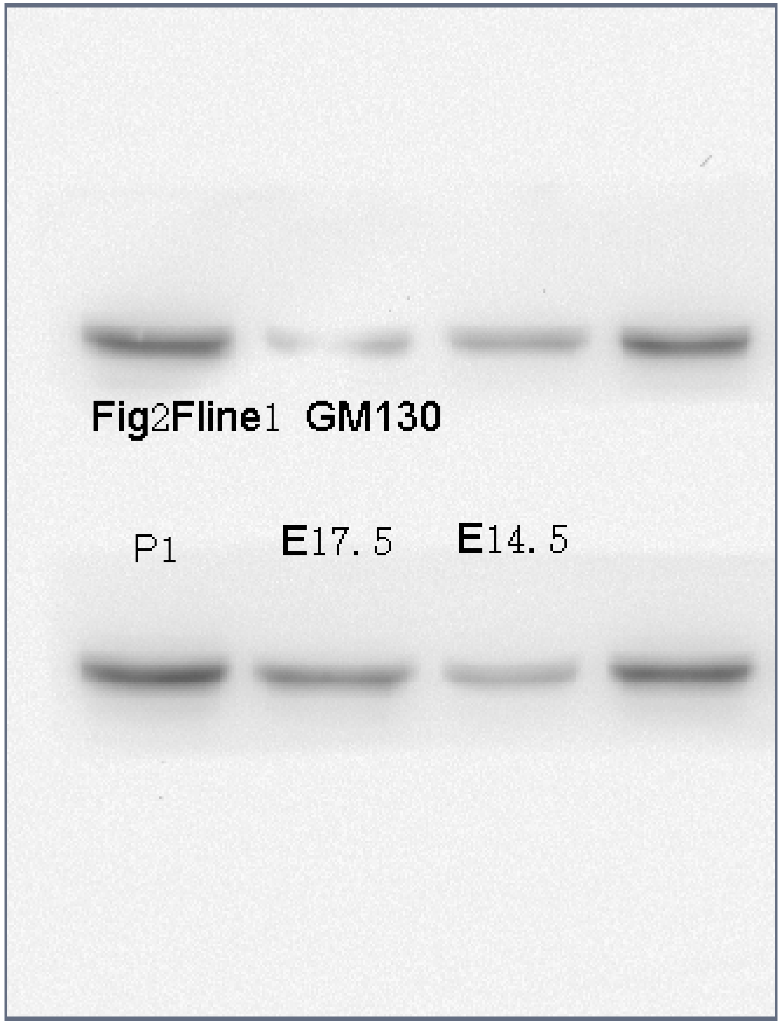
Fig2Fline1 GM130
P1
E17.5
E14.5

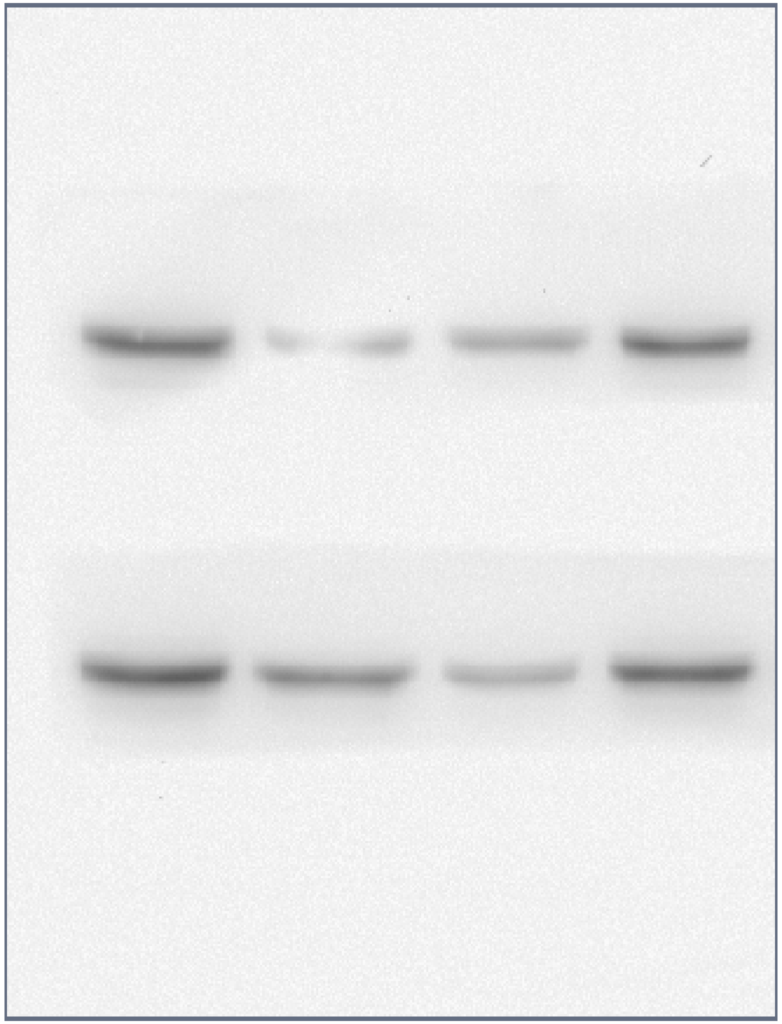

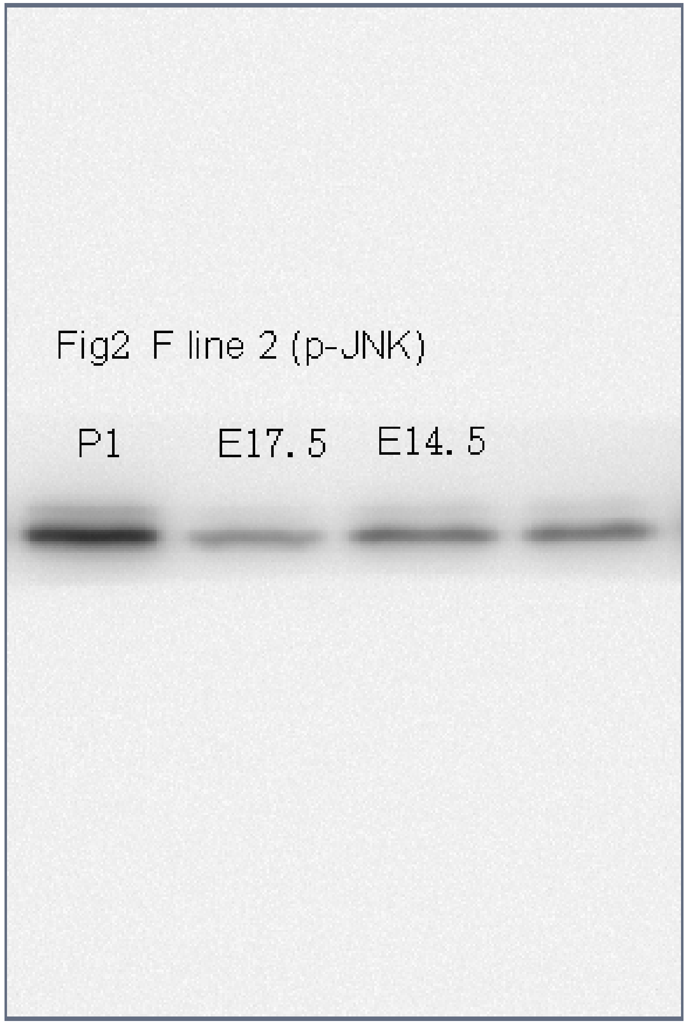
Fig2 F line 2 (p-JNK)
P1
E17.5
E14.5

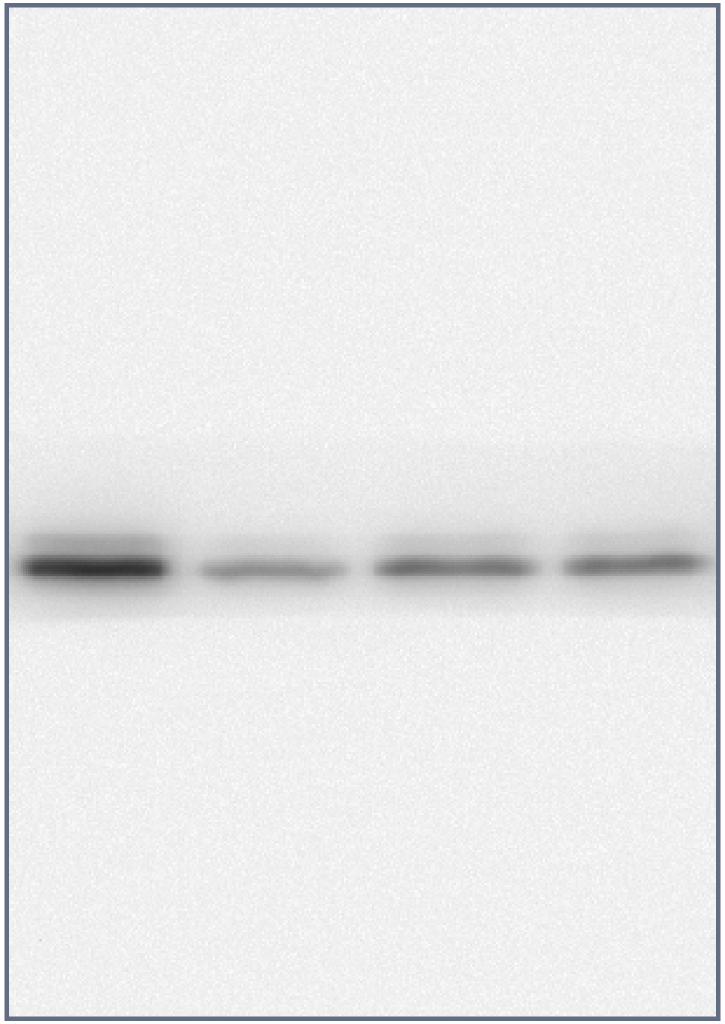

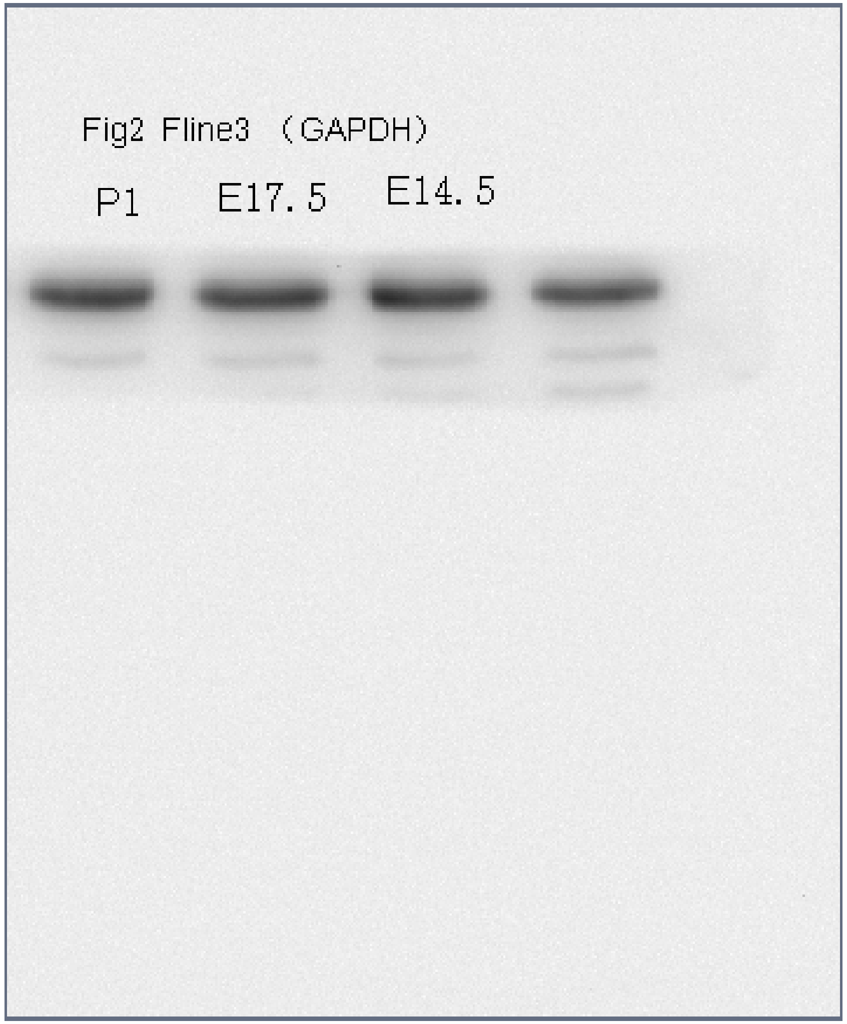
Fig2 Fline3 （GAPDH）
P1
E17.5
E14.5

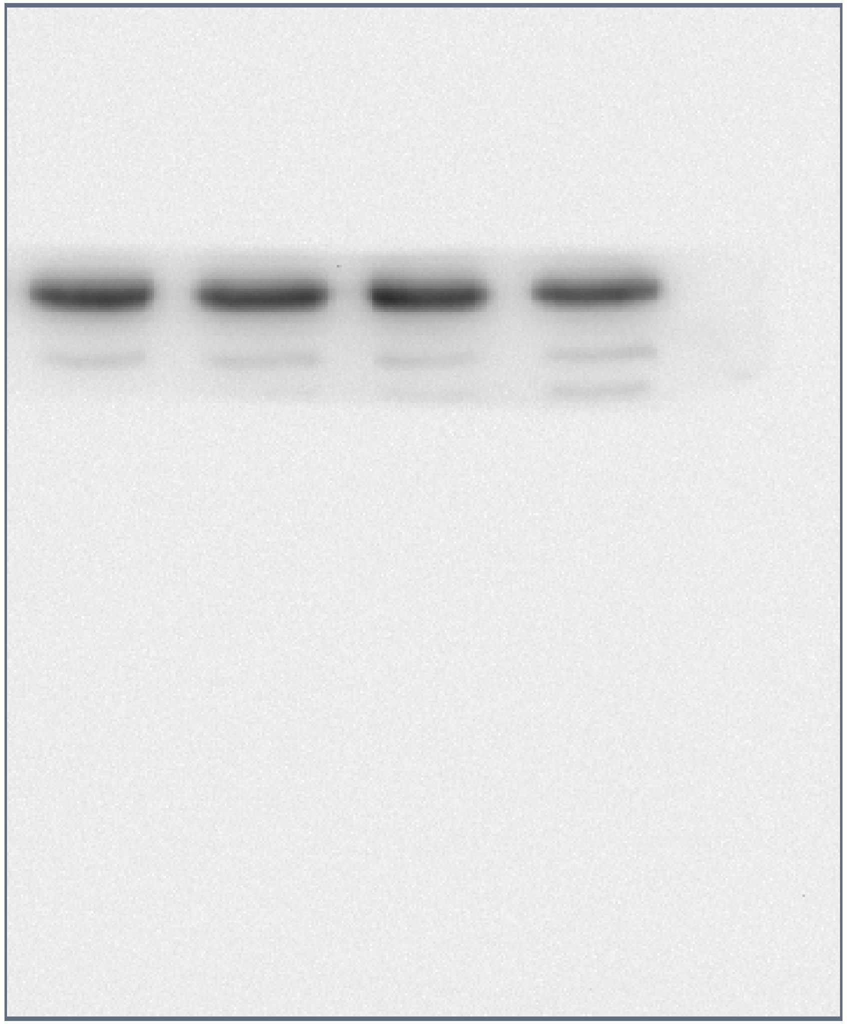

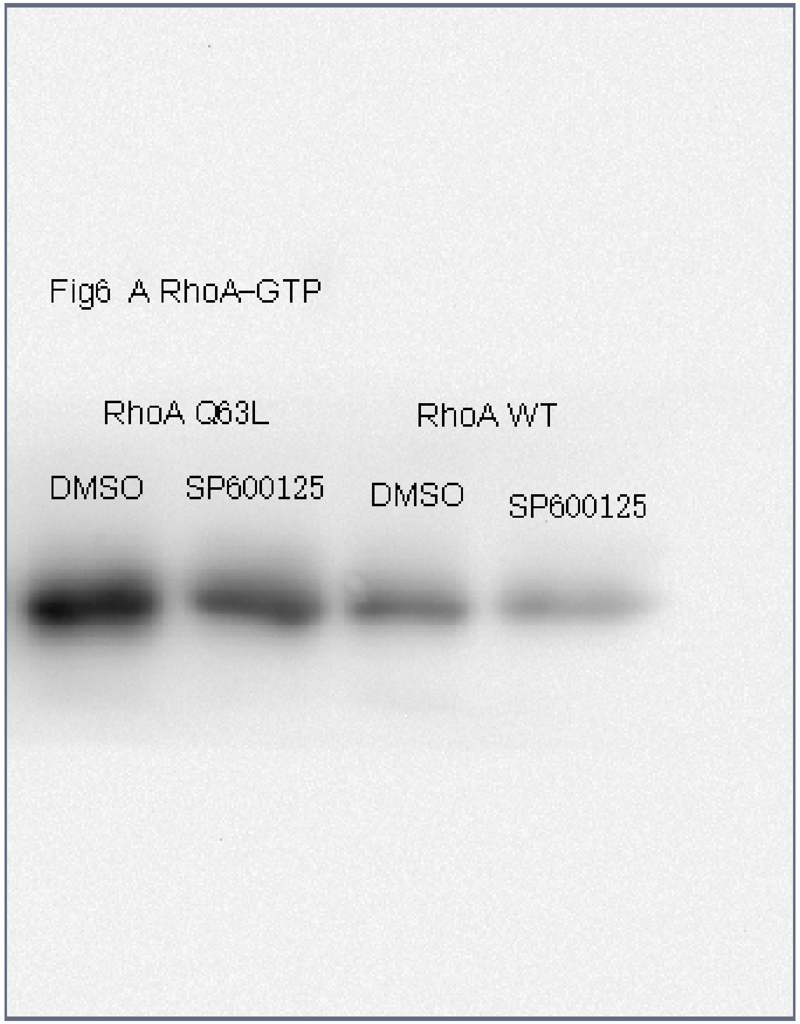

Replicated data

Fig 6 A RhoA-GTP

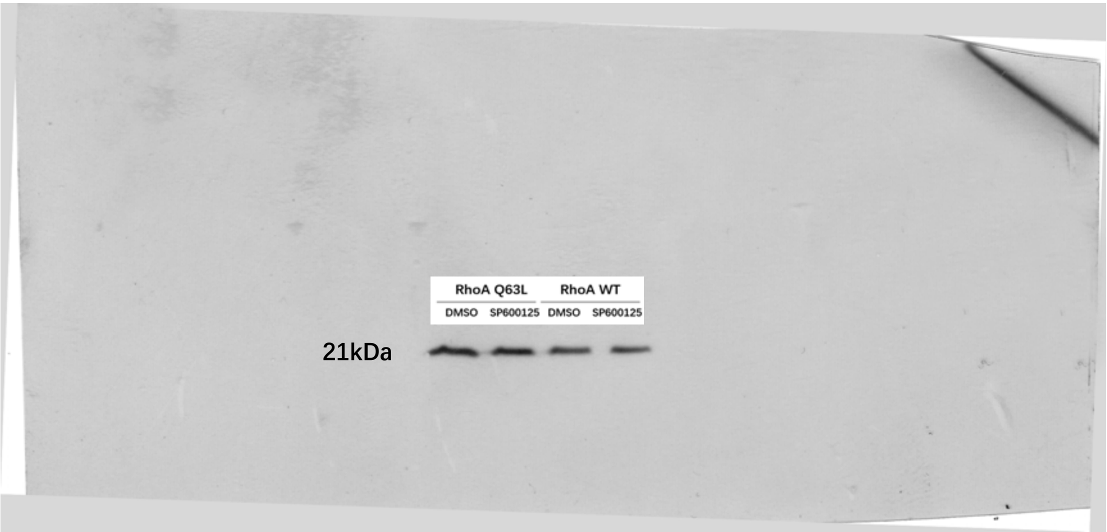

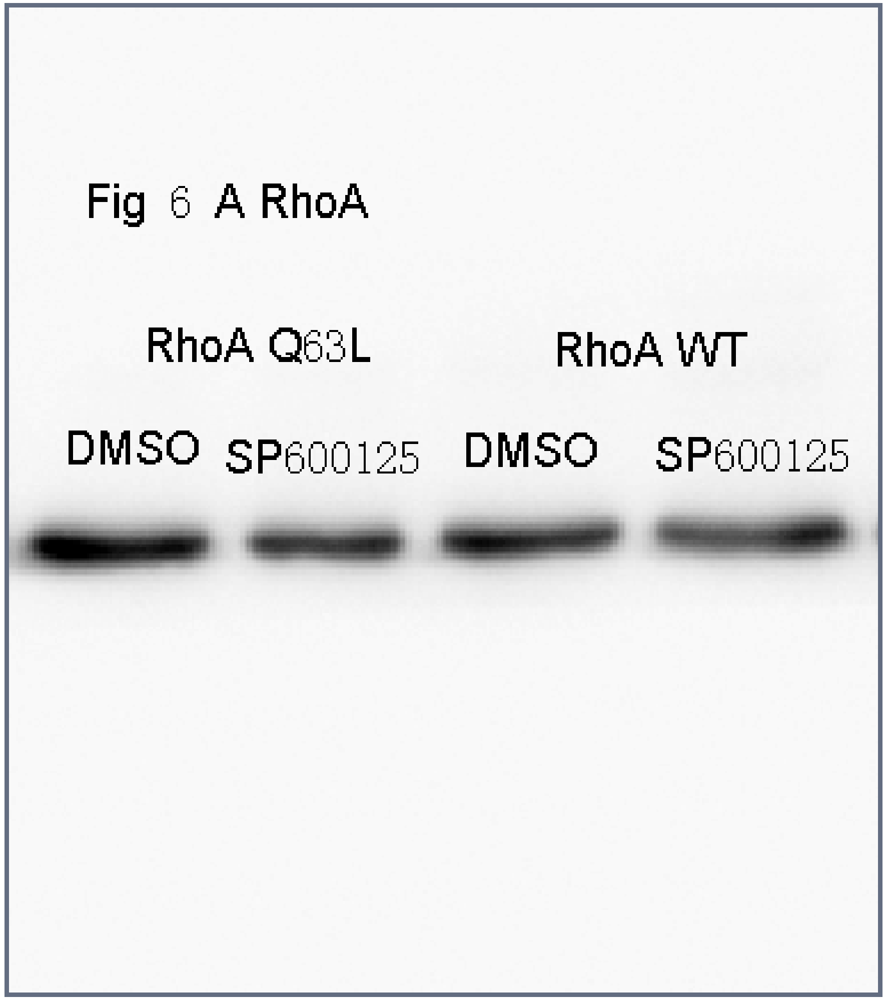

Replicated data

Fig 6 A RhoA

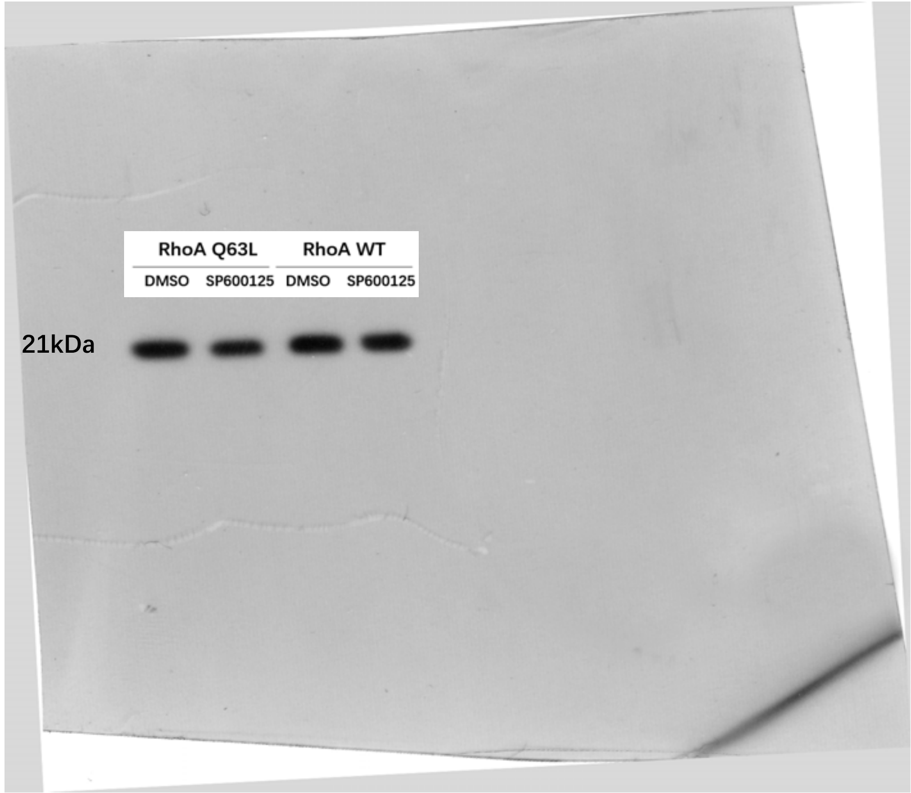

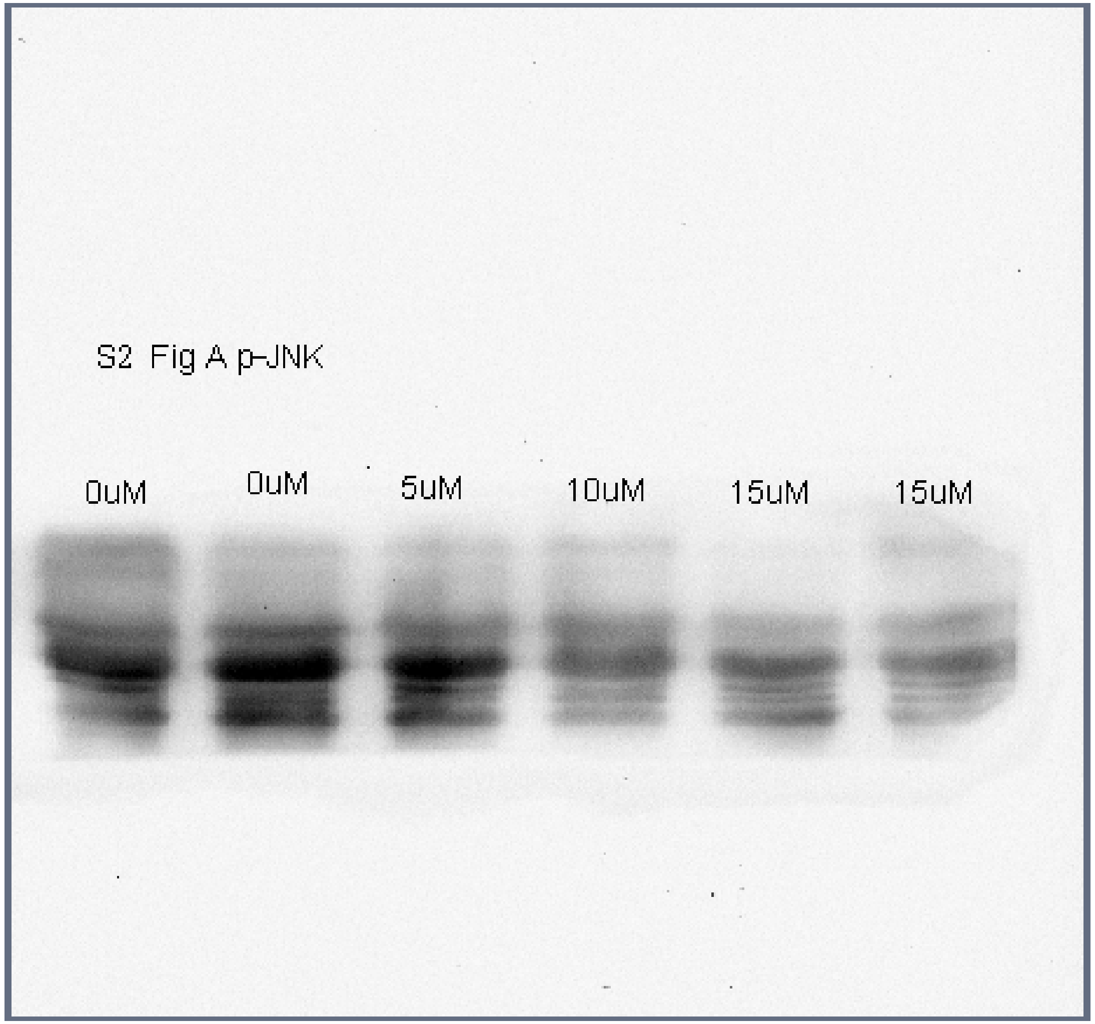

Replicated data

S2 Fig A P-JNK

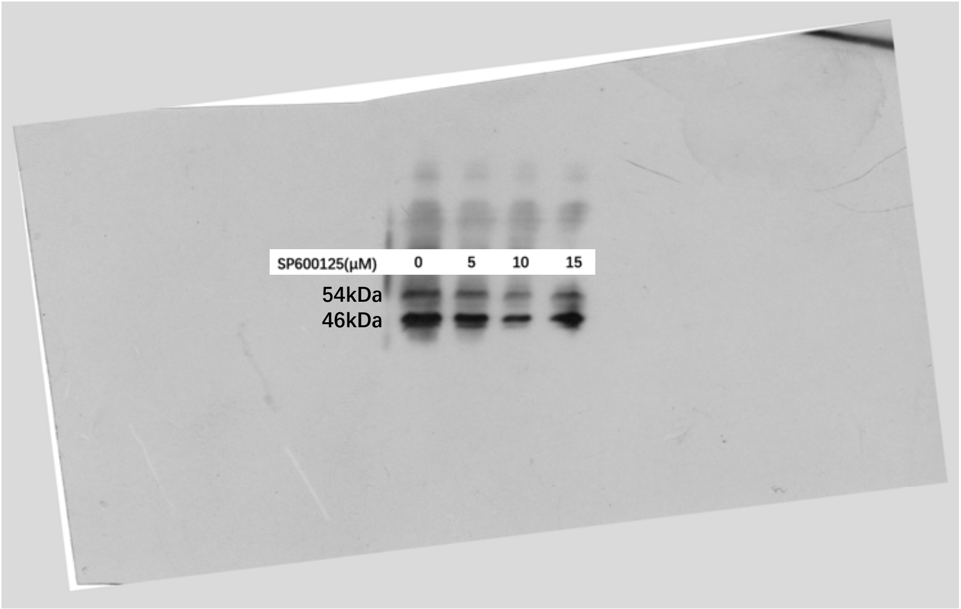

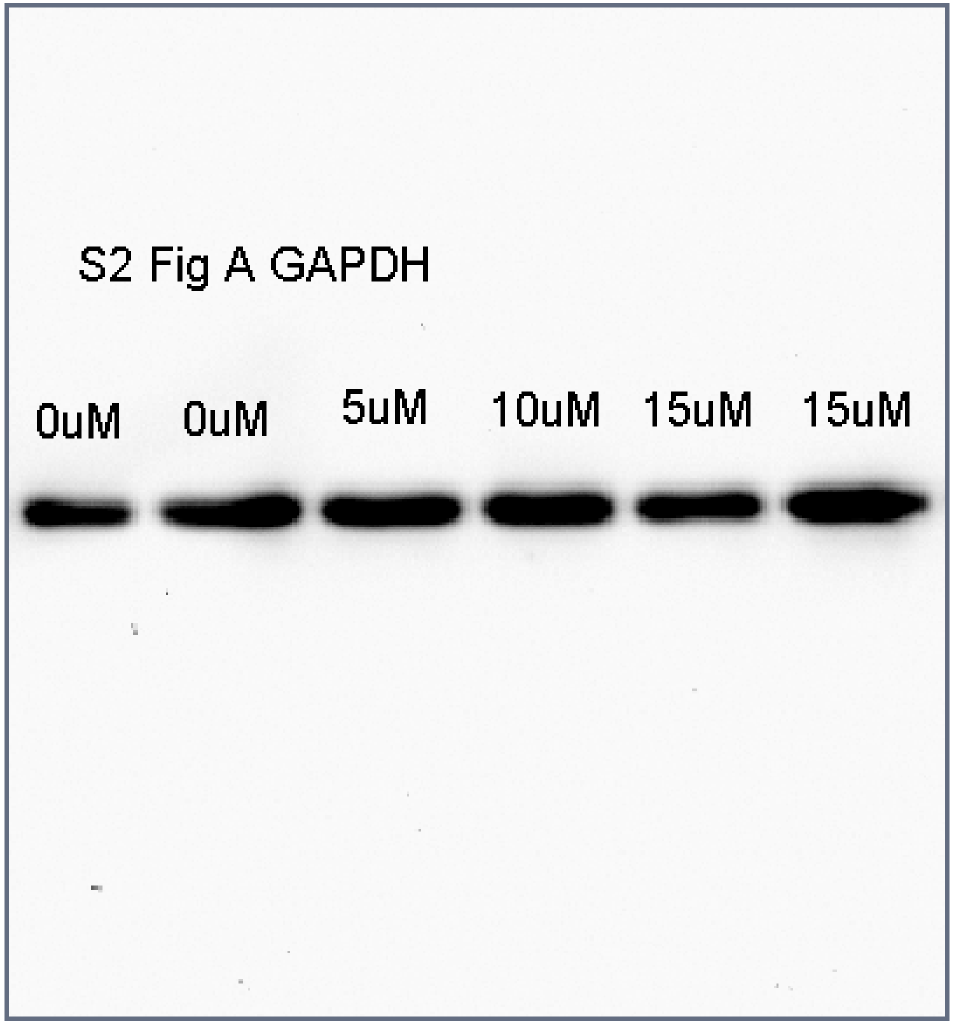

Replicated data

S2 Fig A Total-JNK

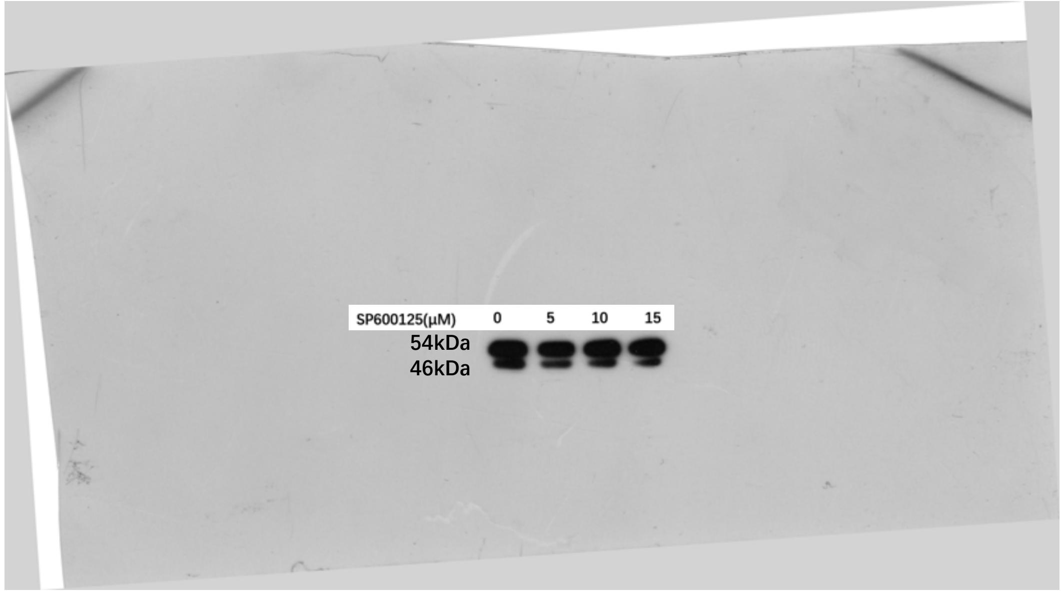

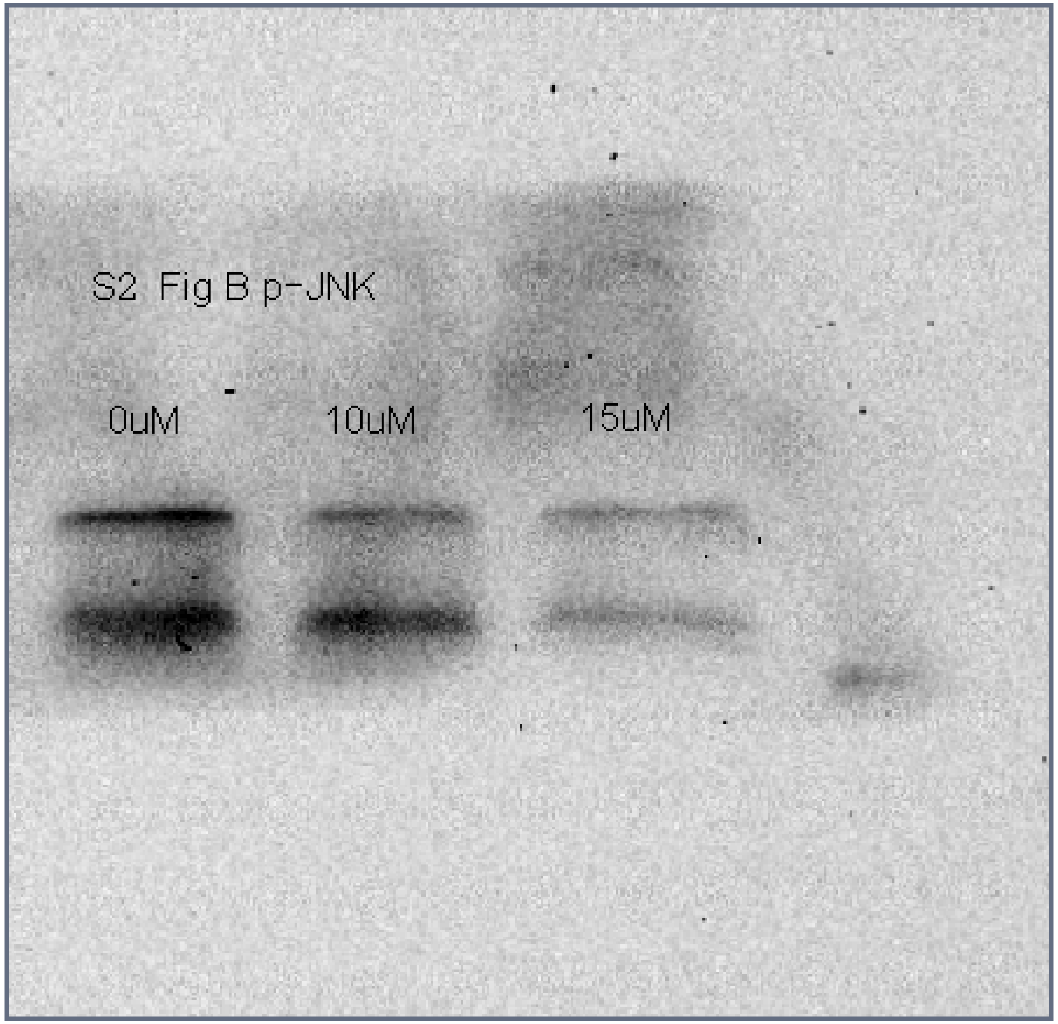

Replicated data

S2 Fig B P-JNK

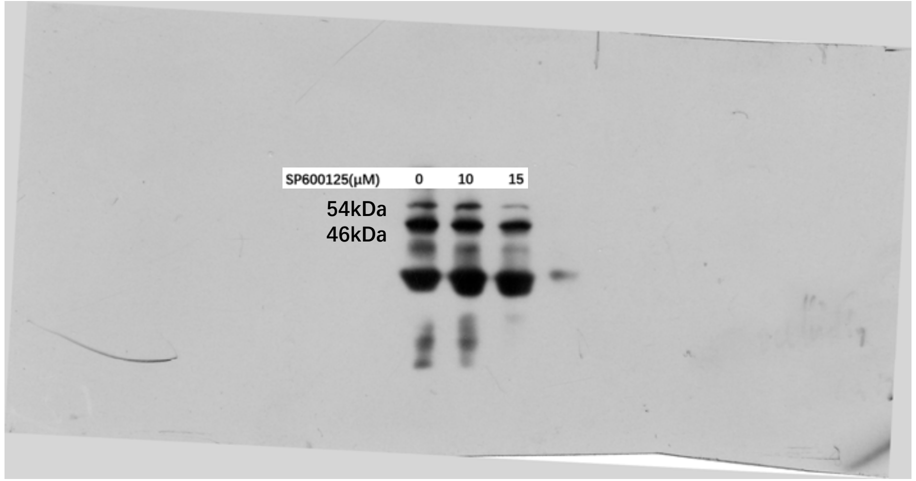

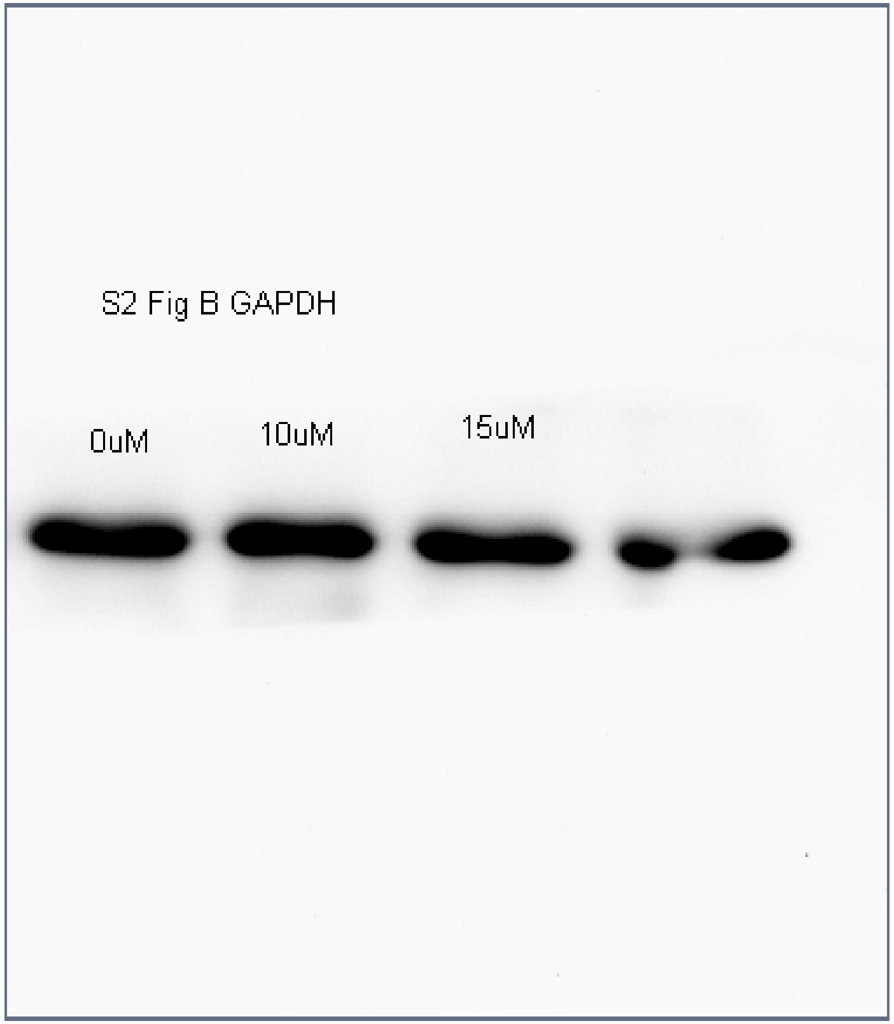

Replicated data

S2 Fig B Total-JNK

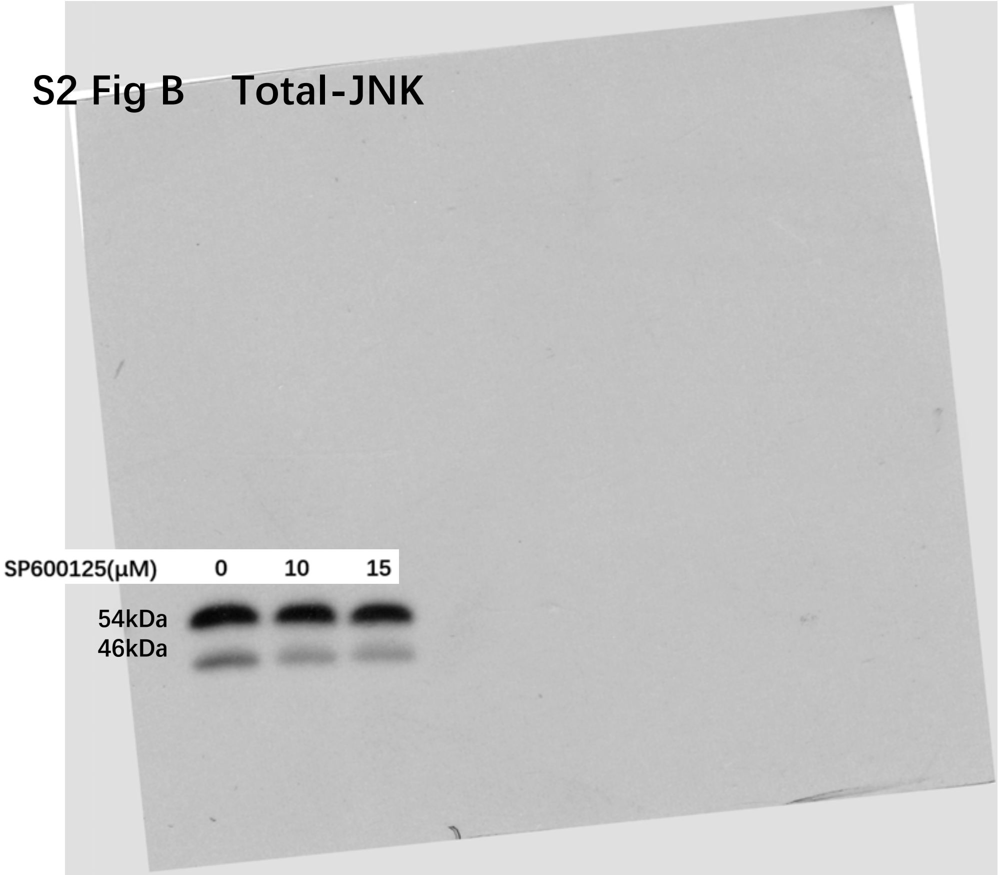

Supplement: S1 Raw images — (PDF) [file pone.0233944.s001.pdf]
