# Supplementary figures and images for: C-Jun N-terminal kinase (JNK) pathway activation is essential for dental papilla cells polarization
Source: PLoS One. 2021 Mar 26;16(3):e0233944. doi: 10.1371/journal.pone.0233944 (PMC7996994; doi:10.1371/journal.pone.0233944)

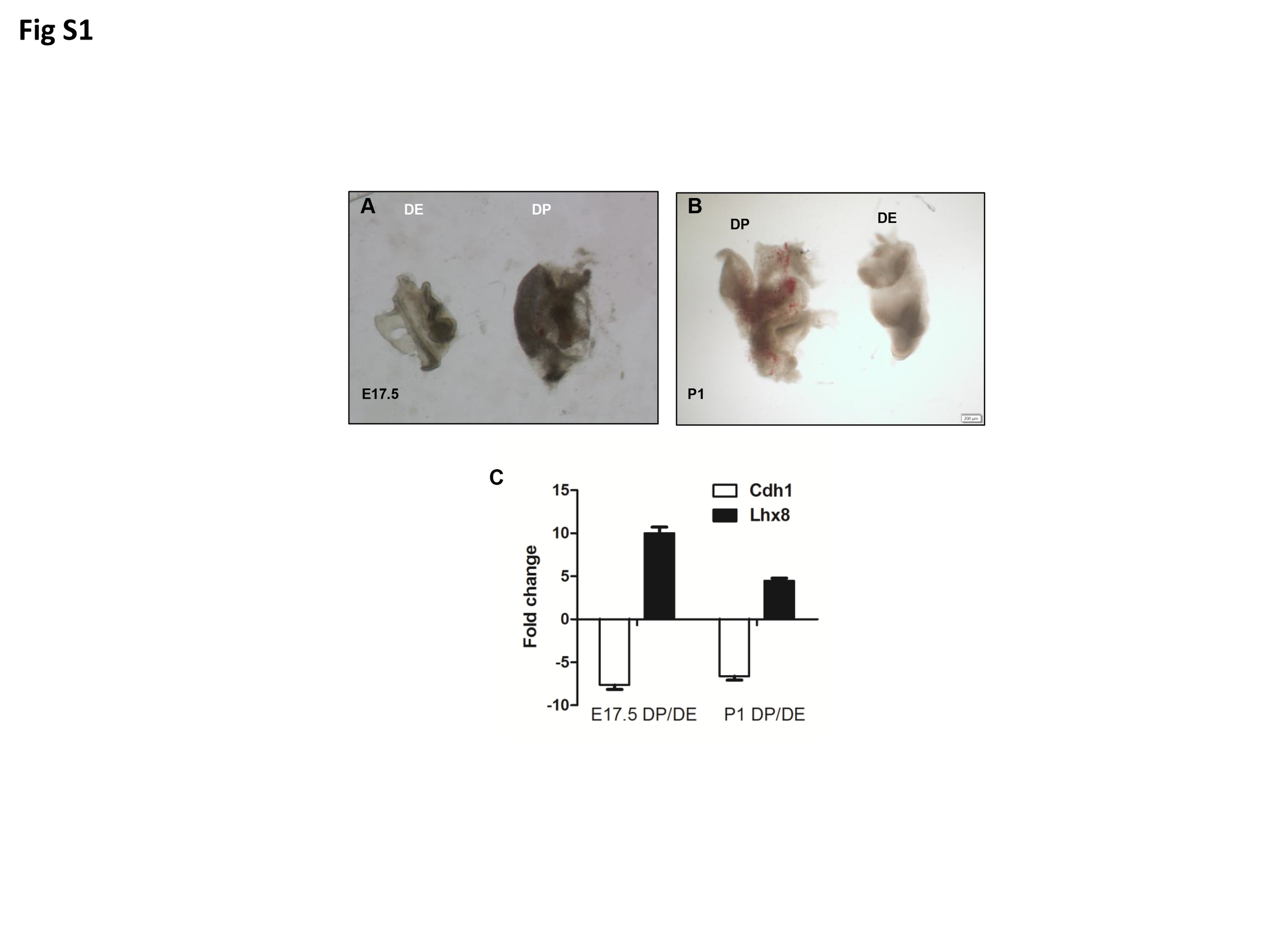

Supplement: S1 Fig — (A, B) Separated dental epithelium and dental papilla under the stereomicroscope. (C) Expression of epithelium marker Cdh1 and mesenchymal marker Lhx8 in the separated dental papilla. DP: dental papilla, DE: dental epithelium. (TIF) [file pone.0233944.s002.tif]

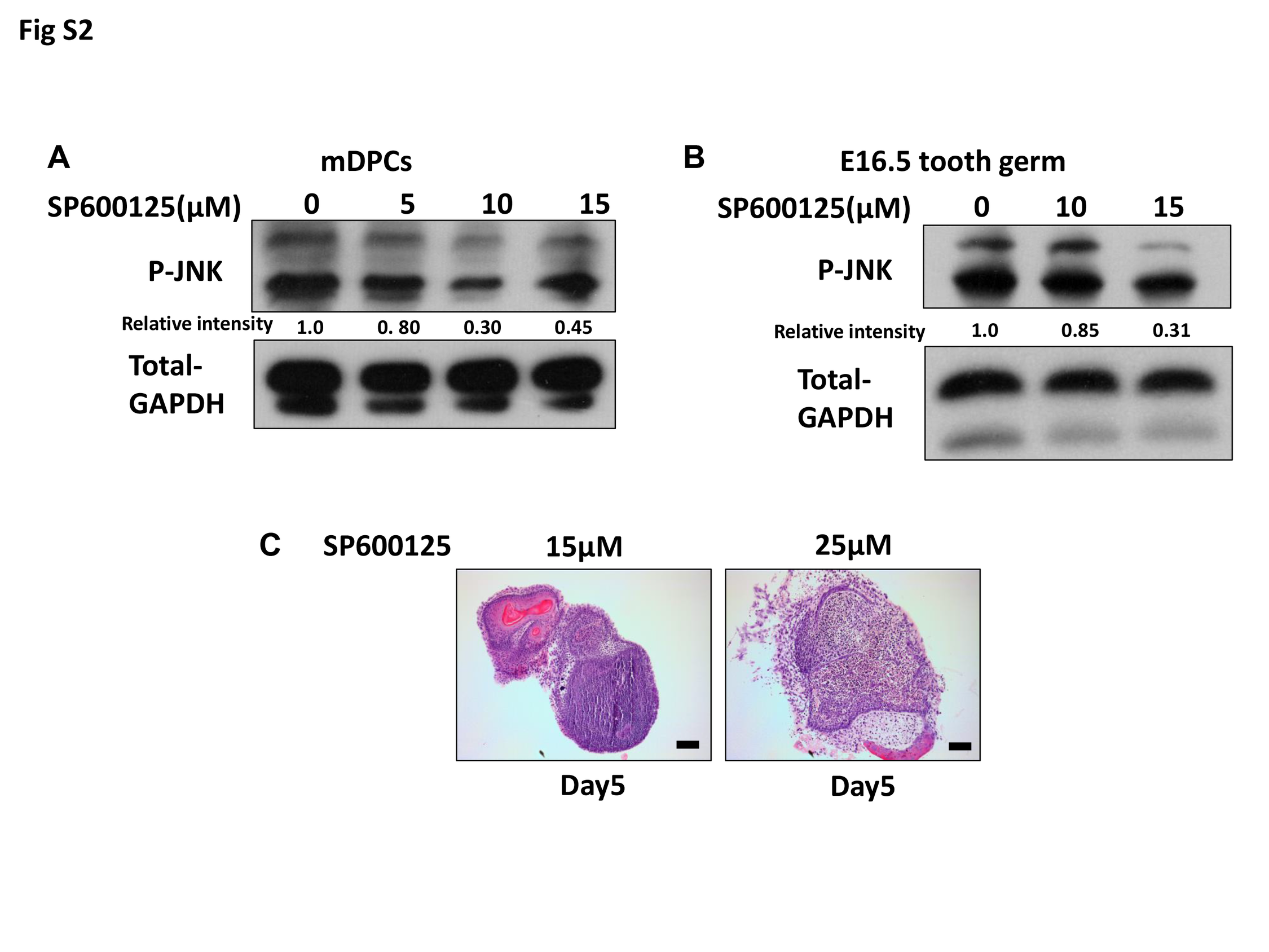

Supplement: S2 Fig — (A) For mDPCs culture, 10μM SP600125 could inhibit JNK signaling activation after 48 hr. (B) For E16.5 tooth germ culture, 15μM SP600125 could inhibit JNK signaling activation after 48 hr. (C) For E16.5 tooth germ culture, 25μM SP600125, but not 15μM SP600125, has some toxic effect on the tissues after 5 days. Bars = 200μm. (TIF) [file pone.0233944.s003.tif]
