# Supplementary material for: C-Jun N-terminal kinase (JNK) pathway activation is essential for dental papilla cells polarization
Source: PLoS One. 2021 Mar 26;16(3):e0233944. doi: 10.1371/journal.pone.0233944 (PMC7996994; doi:10.1371/journal.pone.0233944)
Supplement: S1 Table — (DOCX) [file pone.0233944.s004.docx]

**S1 Table. Primer sequences from Primerbank.**

| **Gene(mouse)** | **S** | **Sequence** | **Product bp** |
| --- | --- | --- | --- |
| **Gapdh** | **F** | TGCACCACCAACTGCTTAGC | 87 |
|  | R | GGCATGGACTGTGGTCATGAG |  |
| **Jnk1** | F | ATGGCTGTCGATATTCAACCAG | 110 |
|  | R | CCTCTTGGGCATACCCCAC |  |
| **Jnk2** | F | TCAGTGGGTTGCATCATGGG | 108 |
|  | R | GGATGGTGTTCCTAGCTGTTCA |  |
| **Cdh1** | F | CAGTTCCGAGGTCTACACCTT | 131 |
|  | R | TGAATCGGGAGTCTTCCGAAAA |  |
| **Lhx8** | F | ACACGAGCTGCTACATTAAGGA | 110 |
|  | R | CCAGTCAGTCGAGTGGATGTG |  |
| **Zeb1** | F | ACTGCAAGAAACGGTTTTCCC | 127 |
|  | R | GGCGAGGAACACTGAGATGT |  |
| **Zeb2** | F | AAACGTGGTGAACTATGACAACG | 245 |
|  | R | CTTGCAGAATCTCGCCACTG |  |
| **Rac1** | F | GAGACGGAGCTGTTGGTAAAA | 138 |
|  | R | ATAGGCCCAGATTCACTGGTT |  |
| **Cdc42** | F | CCAAGACCCCAATTTACCTGAAA | 136 |
|  | R | CCCTCTTTGCCGATGTGTATAGT |  |
| **RhoA** | F | AGCTTGTGGTAAGACATGCTTG | 138 |
|  | R | GTGTCCCATAAAGCCAACTCTAC |  |
| **Map1b** | F | TCGCACCGCTTCCTAGACA | 147 |
|  | R | CTGGTCCAAGTTGCACTCAAT |  |
| **Scrib** | F | GGGGTGATCCAGCCATTGG | 126 |
|  | R | GGCCCTATACGCCTGCTTC |  |
| **Snai1** | F | CACACGCTGCCTTGTGTCT | 133 |
|  | R | GGTCAGCAAAAGCACGGTT |  |
| **Snai2** | F | CATCCTTGGGGCGTGTAAGTC | 186 |
|  | R | GCCCAGAGAACGTAGAATAGGTC |  |
| **Prickle1** | F | ACCTGGAGTATGCTGGCAC | 101 |
|  | R | CACAGTGGATTTTTCCATCCTGA |  |
| **Prickle2** | F | GACAGGAGCTATTTGTGAACAGT | 117 |
|  | R | ACGGTGCATACGAAGCAGG |  |
| **Prickle3** | F | ATGTCAACAGTCCTGGGGAG | 179 |
|  | R | TGGGAAGATTCGAACGGTGG |  |
| **Prickle4** | F | GGCAGATCTCTCCTGTGCTG | 187 |
|  | R | TGGATCTGGCTCCCTGAAGA |  |
| **Golga1** | F | ACAGGTCCGAAACTTGCAGAA | 296 |
|  | R | GCTGGAACCCCTCTAATTCATCA |  |
| **Golga2** | F | GGGCCTCACATCTTCCAACAT | 249 |
|  | R | GACACCAGGATGCCTATGGTC |  |
| **Golga5** | F | CAAATTACCAGACCGGACAGAA | 109 |
|  | R | GATCCGACTTTCACGTTCGCT |  |
| **Golga7**m**b** | F | TGAGGTCCACAATCTTCAGGA | 118 |
|  | R | CTCTGGCGGGAATTTGGTCT |  |
| **Celsr1** | F | ACCAAGGACACTCATGTACTCA | 140 |
|  | R | CGCTCTCGATACTCAGACTGC |  |
| **Vangl1** | F | GATACCGAATCCACGTATTCTGG | 117 |
|  | R | TCTGCCATCTTTATTCCTTGGTG |  |
